# Characterization of Sigma Factor Genes in *Streptomyces lividans* TK24 Using a Genomic Library-Based Approach for Multiple Gene Deletions

**DOI:** 10.3389/fmicb.2018.03033

**Published:** 2018-12-10

**Authors:** Yuriy Rebets, Konstantinos C. Tsolis, Elísabet Eik Guðmundsdóttir, Joachim Koepff, Beata Wawiernia, Tobias Busche, Arne Bleidt, Liliya Horbal, Maksym Myronovskyi, Yousra Ahmed, Wolfgang Wiechert, Christian Rückert, Mohamed B. Hamed, Bohdan Bilyk, Jozef Anné, Ólafur Friðjónsson, Jörn Kalinowski, Marco Oldiges, Anastassios Economou, Andriy Luzhetskyy

**Affiliations:** ^1^Pharmazeutische Biotechnologie, Universität des Saarlandes, Saarbrücken, Germany; ^2^Department of Microbiology and Immunology, Rega Institute, KU Leuven, Leuven, Belgium; ^3^Matis Ohf (MATIS), Reykjavik, Iceland; ^4^IBG-1: Biotechnology, Institute of Bio- and Geosciences, Forschungszentrum Jülich GmbH, Jülich, Germany; ^5^Institute of Biotechnology, RWTH Aachen University, Aachen, Germany; ^6^Center for Biotechnology, Bielefeld University, Bielefeld, Germany; ^7^Department of Molecular Biology, National Research Centre, Giza, Egypt; ^8^Actinobacteria Metabolic Engineering Group, Helmholtz Institute for Pharmaceutical Research Saarland (HIPS), Saarbrücken, Germany

**Keywords:** *Streptomyces lividans*, genomic library, σ-factor, strain phenotyping, transcriptomics, secretomics

## Abstract

Alternative sigma factors control numerous aspects of bacterial life, including adaptation to physiological stresses, morphological development, persistence states and virulence. This is especially true for the physiologically complex actinobacteria. Here we report the development of a robust gene deletions system for *Streptomyces lividans* TK24 based on a BAC library combined with the λ-Red recombination technique. The developed system was validated by systematically deleting the most highly expressed genes encoding alternative sigma factors and several other regulatory genes within the chromosome of *S. lividans* TK24. To demonstrate the possibility of large scale genomic manipulations, the major part of the undecylprodigiosin gene cluster was deleted as well. The resulting mutant strains were characterized in terms of morphology, growth parameters, secondary metabolites production and response to thiol-oxidation and cell-wall stresses. Deletion of SLIV_12645 gene encoding *S. coelicolor* SigR1 ortholog has the most prominent phenotypic effect, resulted in overproduction of actinorhodin and coelichelin P1 and increased sensitivity to diamide. The secreted proteome analysis of SLIV_12645 mutant revealed SigR1 influence on trafficking of proteins involved in cell wall biogenesis and refactoring. The reported here gene deletion system will further facilitate work on *S. lividans* strain improvement as a host for either secondary metabolites or protein production and will contribute to basic research in streptomycetes physiology, morphological development, secondary metabolism. On the other hand, the systematic deletion of sigma factors encoding genes demonstrates the complexity and conservation of regulatory processes conducted by sigma factors in streptomycetes.

## Introduction

Actinobacteria are large group of soil-dwelling bacteria occupying diverse ecological niches, ranging from different types of soils to the specialized glands of insects (Scott et al., 2008; Kaltenpoth et al., 2012). Such habitat diversity entails that these bacteria are facing an enormous diversity of conditions that are not always ideal for growth. Furthermore, these conditions often change rapidly, imposing new challenges for bacterial survival. This led to the development in Actinobacteria of a highly branched regulatory network sensing environmental changes and responding by re-tuning metabolic pathways, modulating growth rates and activating morphological processes (Bruijn, 2016). These regulatory networks mainly act by mediating transcription of specific sets of genes. Thus it is not surprising that actinobacterial genomes are enriched in genes coding for alternative RNA polymerase sigma factors and diverse families of transcriptional regulators (Bentley et al., 2002; Ruckert et al., 2015).

Sigma factors are essential regulatory subunits of the bacterial RNA polymerase and confer promoter specificity to the catalytic core RNA polymerase. They specifically recognize critical promoter elements, thus directing expression of specific sets of genes (referred to as “regulons” of the corresponding sigma factor). All bacteria contain essential primary sigma factors, directing the expression of house-keeping genes and one or more non-essential, alternative sigma factors having diverse roles (e.g., in responding to various stresses and cell differentiation) (Paget, 2015). For instance, two model bacteria, *Escherichia coli* and *Bacillus subtilis*, contain 7 and 18 different sigma factors, respectively (Gruber and Gross, 2003). Sigma factors can be classified into two functionally, structurally, and phylogenetically distinct families, σ^70^ and σ^54^. The dominant σ^70^ family is further subdivided into four groups; (1) essential primary sigma factors directing expression of most house-keeping genes; (2) non-essential homologues of primary sigma factors; (3) alternative sigma factors; (4) extracytoplasmic function (ECF) sigma factors. The alternative sigma factors are further subdivided into four functionally related subgroups: (1) flagellar sigma factors; (2) heat shock sigma factors; (3) general stress response sigma factors; (4) sporulation sigma factors. Alternative sigma factors are regulated at the transcriptional, translational, and post-translational levels. One common mechanism of their regulation is a reversible interaction with their specific negative regulators, anti-sigma factor proteins. The latter sequester sigma factors and prevent their interaction with core RNA polymerase (Gruber and Gross, 2003; Paget, 2015).

All streptomycetes contain a large linear chromosome (Bentley et al., 2002). The 8.7 Mb genome of the best-studied model organism *Streptomyces coelicolor* A3(2) contains 66 chromosomal sigma factor-encoding genes, a non-functional truncated one and three located on the linear plasmid SCP1 (Paget et al., 2002; Bentley et al., 2004). They include one principal sigma factor HrdB (SCO5820); 3 non-essential homologs of principal sigma factors: HrdA (SCO2465), HrdC (SCO0895), and HrdD (SCO3202), with currently unknown roles; a homolog of the flagellar sigma factor: WhiG (SCO5621), controlling aerial mycelium septation; 9 close homologs of the *B. subtilis* general stress-response sigma factor SigB (SigB,F,G,H,I,K,L,M,N) which have functions mainly in osmotic stress response and morphological differentiation; and 51 ECF sigma factors (Paget et al., 2002). Only a few of the ECF sigma factors have been characterized in *S. coelicolor* A3(2) to date. One of these, SigE (SCO3356), has a role in cell wall homeostasis (Paget et al., 1999); SigQ (SCO4908) and SCO4117 are required for both secondary metabolism and differentiation (Lopez-Garcia et al., 2018); SigR (SCO5216) has a role in thiol-oxidative stress response (Paget et al., 1998); BldN (SCO3323) has a role in morphological differentiation (Bibb et al., 2000); LitS (SCO0194) is required for light-induced production of carotenoids (Takano et al., 2005); SigT (SCO3892) and SigU (SCO2954) negatively regulate morphological differentiation, secondary metabolism and positively control protein secretion (Gehring et al., 2001; Mao et al., 2009). The significance of sigma factors in control of different physiological processes in Actinobacteria is reviewed by Sun et al. (2017).

Although several sigma factor regulons have been characterized in *S. coelicolor* A3(2), no study has specifically focused on their role in protein secretion. Several members of the SigB osmotically induced regulon (SCO2342, SCO2591, SCO3097, SCO4471, SCO7646, SCO7657) have only been defined as secreted proteins (Lee et al., 2005). One member of the SigR regulon (SCO7631) has also been annotated as a secreted protein (Kim et al., 2012). Study related to SigU suggested some role of this sigma factor in protein secretion in *S. coelicolor* (Gordon et al., 2008). The regulon of this sigma factor contains several annotated secreted proteins, including secreted proteases (SCO0732, SCO0752, SCO0930, SCO1356, SCO2217, SCO0644, SCO0986, SCO1573, SCO2207). A *S. coelicolor* strain with high SigU activity (due to its anti-sigma factor gene *rsuA* being deleted), secreted a much greater quantity and diversity of proteins, including many secreted proteases, than the wild-type *S. coelicolor*. Conversely, the secreted protease inhibitor SCO0762 was found in much reduced amounts in the mutant, resulting in increased extracellular protease activity.

*Streptomyces lividans* TK24 is a species closely related to *S. coelicolor* M145 with only 507 genes being different (Ruckert et al., 2015). *S. lividans* TK24 was derived from *S. lividans* strain 66 after UV mutagenesis and protoplast regeneration and, like its parental strain, is producing actinorhodin, the calcium-dependent antibiotic and undecylprodigiosin only under certain growth conditions and with a low titer (Hopwood et al., 1983). Due to low endogenous protease activity and reproducible growth behavior during cultivation in constant environmental conditions, *S. lividans* TK24 is an excellent host strain for heterologous secretion of proteins (Anné et al., 2017; Koepff et al., 2018). Despite being used for decades as a host for secondary metabolites and protein production, in basic research *S. coelicolor* was preferred over *S. lividans* strains. Typically, the knowledge obtained on *S. coelicolor* was extrapolated to *S. lividans* without verification. This is also true for the functions of sigma factor- encoding genes for which no data in *S. lividans* exist.

The last decade in genetics of Actinobacteria resulted in rapid development of tools for genome modifications and engineering (Rebets et al., 2014). Such tools, accelerating the genome manipulations in *Streptomyces*, are especially valuable due to the importance of these bacteria and their physiological peculiarities such as slow growth. These techniques are based on recombination and allow rapid generation of multiple deletions within the same strain by re-utilization of resistance markers (Myronovskyi et al., 2014), quick selection of secondary cross-over events (Myronovskyi et al., 2011; Knirschova et al., 2015), precise introduction of point mutations in desired sites or removal of large portion of a genome (Cobb et al., 2015; Lopatniuk et al., 2017). Progress in tool development reached the point, at which mass deletion of genes in the same strain has become a routine procedure. The indispensable components of many of these procedures are arranged genomic libraries of the strain of interest.

Here, we report the construction of a *S. lividans* TK24 genomic library based on a *gusA*-containing (encoding β-glucuronidase) BAC vector. This allows for simple and fast selection of gene deletion strains and its utilization in combination with the IMES [Iterative Markers Excision System (Myronovskyi et al., 2014)] antibiotics resistance cassettes for systematic inactivation of sigma factors and regulatory genes within the chromosome of *S. lividans* TK24. Obtained mutants were characterized in terms of growth, morphology, stress-tolerance and protein secretion, revealing the complex regulatory functions of the deleted genes on the physiology of *S. lividans* TK24.

## Materials and Methods

### Bacterial Strains, Culture Conditions and General Procedures

*Streptomyces* strains were grown on solid nutrient medium MS (mannitol soy flour agar), minimal medium and in liquid or solid TSB medium (Kieser et al., 2000). For secondary metabolite production NL19 (MS medium without agar), SG and R5A media were used (Kieser et al., 2000). For pre-culture *Streptomyces* strains were inoculated from spores (∼10^8^–10^9^) into 10 ml of TSB medium in 100 ml Erlenmeyer flasks with 1 g of glass beads (5 mm diameter, Sigma Aldrich, United States) and incubated on rotary shaker at 220 rpm and 28°C for 2 days. 2 ml of pre-culture was used to inoculate 50 ml of production medium in 500 ml Erlenmeyer flask with 5 g of glass beads (5 mm diameter, Sigma Aldrich, United States) and incubated on rotary shaker at 220 rpm and 28°C for 5 days. On solid medium *Streptomyces* strains were inoculated from spores and incubated at 28°C for 5–7 days. MS (mannitol soy flour agar), Minimal medium and TSB supplemented with agar were used for quick strain phenotyping (Kieser et al., 2000). *Escherichia coli* XL1Blue (Agilent, United States) was used for routine cloning, and *E. coli* ET12567 (pUB307) was used as a donor in the intergenic conjugation (Flett et al., 1997). *E. coli* strains were grown in Luria-Bertani (LB) broth at 37°C. When required, antibiotics were added to the cultures at the following concentrations: 50 μg ml^−1^ apramycin, 100 μg ml^−1^ hygromycin B, 100 μg ml^−1^ phosphomycin, 100 μg ml^−1^ carbenicillin and 35 μg ml^−1^ kanamycin (Sigma Aldrich, United States; Roth, Germany).

### Lab-Scale Bioreactor Cultivation

Lab-scale cultivations of *S. lividans* were carried out in parallelized glass bioreactors (DasGip, Jülich, Germany) with a working volume of 1000 mL. A minimal medium supplemented with 10 g/L D-glucose and 5 g/L Bacto^TM^ Casamino acids was used (D’Huys et al., 2011). Pre-culture conditions were identical to the described in Koepff et al. (2017). A constant pH of 6.8 was maintained by titrating 4 M NaOH or 4 M HCl solutions if required. Detailed procedures for reactor setup and sample generation were performed as published (Koepff et al., 2017).

### Transcriptomics, Identification of Highly Transcribed Genes

Samples (10 ml of bioreactor grown culture) were taken during the early and late log growth phase as well as after entry into the stationary phase. Harvesting and RNA isolation was performed as described previously (Busche et al., 2012). Samples from three biological replicates were isolated separately and pooled after quality control. The RNA quality was checked via Agilent 2100 Bioanalyzer (Agilent Technologies, Böblingen, Germany) and Trinean Xposesystem (Gent, Belgium) prior and after rRNA depletion using the Ribo Zero rRNA Removal Kit for Bacteria (Epicentre, Madison, WI, United States). The RNAseq procedure is described in Busche et al. (2018). The TruSeq Stranded mRNA Library Prep Kit from Illumina was used to prepare the cDNA libraries, which were then sequenced in paired-end mode on an Illumina HiSeq 1500 system with 28 respectively 70 bases read length.

Transcripts per kilobase million (TPM) were calculated using READXPLORER v.2.2 (Hilker et al., 2016). For differential RNA-Seq analyses the signal intensity value (*A*-value) was calculated by average log_2_(TPM) of each gene and the signal intensity ratio (*M*-value) by the difference of log_2_(TPM). In cases where the TPM for a gene was 0, a TPM of 0.1 was used instead to avoid log_2_(0). To identify proteases that were strongly transcribed and differentially expressed under at least one condition, the RNA-Seq data were filtered using a TPM cut-off of 100 and an *M*-value cut-off of > 1.0 under at least one condition.

### Construction of *S. lividans* TK24 Genomic BAC Library

*Streptomyces lividans* was cultivated in Phage medium for approximately 16 h (Rebets et al., 2017), cells collected by centrifugation and DNA extracted using the QIAGEN Genomic-tip 100/G method. To obtain fragments of appropriate size, DNA was electrophoresed on Agarose-HEPES gels with or without prior fragmentation using HydroShear DNA shearing system (DigLab) with 10 cycles at speedcode 40, and fragments of 40kb or greater purified from low melting point agarose using phenol:chloroform extraction. Fragment end repair of the recovered DNA was done using the Lucigen DNATerminator End Repair Kit. Non-palindromic adapters, containing *Bst*XI overhangs (Life Technologies), were then added to the inserts by T4 ligation.

Vector pSMART BACgus was cut with *Bst*XI and ligated with the *Bst*XI adapted inserts in various ratios using T4 ligase. Transformation was carried out with electroporation using BAC-optimized Replicator v2.0 Electrocompetent Cells from CopyRight v2.0 BAC Cloning Kit (Lucigen). The cells were grown on YT-agar with chloramphenicol at 37°C overnight. An insert:vector fragment end ratio of 1:1 – 2:1 resulted in the highest titer of clones.

Single colonies were picked to inoculate 100 μL L-broth with chloramphenicol and grown at 37°C overnight. These cultures were then used to inoculate 1.5 mL cultures of L-broth with chloramphenicol for selection and arabinose to increase the plasmid copy number [Lucigen BAC Cloning Kit; (Wild et al., 2002)] and grown at 37°C for approximately 20 h. Plasmid isolation was done using the NucleoSpin 96 Flash method (Macherey-Nagel) and the plasmid DNA subsequently cut with *Hind*III overnight and run on PFGE/standard agarose gels to confirm the presence and size of inserts. A total of 4465 BAC clones were analyzed on agarose gels with regard to insert size.

Plasmids with large inserts (15–60 kb) were end-sequenced with the following primers binding to the vector sequence flanking the insert: SL1: 5′-CAGTCCAGTTACGCTGGAGTC-3′ sequencing into the 5′ end and SR4: 5′-TTGACCATGTTGGTATGATTT-3′ sequencing into the 3′ end. Sequencing was done using BigDye terminator cycle sequencing chemistry and ABI 3750 DNA sequencer (PE Applied Biosystems). Where sequences of sufficient quality could not be obtained by sequencing plasmid templates, four PCR amplifications using either SL1 or SR4 primers, paired with either GC rich arbitrary primer Arb2: GGCCACGCGTCGACTAGTACNNNNNNNNNNACGCC or AT rich primer Arb1: GGCCACGCGTCGACTAGTACNNNNNNNNNNGATAT. If no clear bands were detected, second nested PCRs were performed with SL1 and SR4 and ARB 3: GGCCACGCGTCGACTAGTAC. The PCR products were then sequenced with ABI Sanger sequencing chemistry.

A total of 2877 clones were subjected to sequencing, sequence information from both ends of 15–100 kb inserts were obtained for 1889 clones. Sequences were mapped to the *S. lividans* genome using Geneious version R9^1^ (Kearse et al., 2012) to identify the location of each clone in the genome. A total of 1632 clones covering 96.4% of the genome were stored in 17 × 96 well plates, in L-broth with 20% glycerol at −80°C. All BAC clones are available on request from MATIS (Iceland).

### Generation of Mutant Strains

To delete selected genes, BAC clones from ordered *S. lividans* BAC library were selected and mutagenized using the λ-Red recombination technique combined with the apramycin or hygromycin resistance IMES cassette from patt-saac-oriT or patt-shyg-oriT plasmids, respectively (Myronovskyi et al., 2014). Primers used to amplify the cassette and to verify the mutation are listed in Supplementary Table 1. λ-Red recombineering was performed as described (Gust et al., 2004). The resulting recombinant BACs were introduced into *S. lividans* TK24 via conjugation (Flett et al., 1997). Screening for double-crossover mutants was performed on MS medium supplemented with 50 μg ml^−1^ of apramycin or 100 μg ml^−1^ hygromycin B and 70 μg ml^−1^ of X-gluc (X-gluc Direct, Spain). Gene deletions were confirmed by PCR using appropriate primers (Supplementary Table 1).

### Microtiter-Plate Based Screening for Rapid Strain Phenotyping

Cultivations, data collection and analysis procedures were as described (Koepff et al., 2017). Spores harvested from MS plates were used to prepare deep-frozen mycelium aliquots of all strains. If needed, respective vials were thawed and used to inoculate magnetically stirred Erlenmeyer pre-culture flasks, pre-filled with complex cultivation medium (Kieser et al., 2000). Optical density of pre-cultures was determined to adjust the inoculation density for main-culture cultivations to a final OD600 of 0.2 (UV1800, Shimadzu, Kyoto, Japan). Main cultures (1 mL) were conducted in biological triplicates in 48-well microtiter-plates incubated in an automated cultivation device (m2p-labs, Baesweiler, Germany) at 30°C and 900 rpm shaking frequency. In this step, a minimal medium supplemented with 10 g/L D-glucose, 5 g/L Bacto^TM^ Casamino acids and pH-buffered to 7.2 using 100 mM MES was used (D’Huys et al., 2011). For technical reasons, not all strains were cultivated in the same microtiter-plate. However, to compensate possible batch-related differences the wild-type strain TK24 was cultivated in all plates and all other obtained values were normalized on the corresponding wild-type results. The light back-scattering was used to track the growth of cultures; oxygen and glucose consumption were monitored online as described (Koepff et al., 2017). Final dry cell mass was determined by filtration as described (Koepff et al., 2017). The pellets size was determined by light microscopy. The four parameters [cell dry weight (CDW), maximum scattered light, maximum specific growth rate (μ_*max*_) and average pellet size] considered for strain phenotyping, were obtained by a standardized workflow (Supplementary Figure 1).

### Analysis of Diamide, Hydrogen Peroxide, and Vancomycin Sensitivity

Sensitivity of the *S. lividans* mutants to diamide and vancomycin was tested by plating 100 μL of spore suspension (∼10^9^/mL) of each strain on fresh minimal medium, MS or TSB agar plates. Plates were dried for 10 min. Paper disks loaded with 5 or 10 μM of diamide (aqueous solution), 250 and 500 μg of vancomycin (aqueous solution) or soaked in 10% solution of hydrogen peroxide were placed on inoculated plates, and cultures growth was analyzed after 3 days of incubation at 30°C. Disks with diamide and vancomycin were pre-dried before usage. The tests were repeated three times for each strain (technical replicates). The inhibition zones were measured.

### Secretomics Sample Preparation and Measurement

For secretomics analysis strains were cultivated in 48-well microtiter-plates with monitoring cell growth by light back-scattering and oxygen and glucose consumption as described above in Section “Microtiter-Plate Based Screening for Rapid Strain Phenotyping.” Cells were harvested after 24 h when cultures reached stationary phase of growth (Koepff et al., 2017). Cells were removed by centrifugation (4,500 × g; 5 min; 4°C) and subsequent filtration (syringe filter, 0.2 μm, cellulose acetate; Corning, NY, United States). Proteins contained in culture supernatants were precipitated via 25% w/v TCA precipitation (4°C; 20 min). Precipitated proteins were pelleted via centrifugation (20,000 × g; 20 min; 4°C), on a bench-top centrifuge. The pellet was washed twice with ice-cold acetone and re-pelleted via centrifugation (20,000 × g; 20 min; 4°C). The protein pellet was then solubilized in 8M urea in 1M ammonium bicarbonate solution (ABS). Polypeptide concentrations were measured using the Bradford reagent. Polypeptides (3 μg) were separated by SDS-PAGE on 12% polyacrylamide gel and visualized by silver staining (Shevchenko et al., 1996).

### Analysis of Secretome by Nano LC-MS/MS

A volume corresponding to the secreted polypeptides derived from 3 × 10^6^ cells (usually a volume equivalent to 20–40 μL of the initial cell culture) was used for in-solution tryptic digestion. The protein solution was initially diluted into urea (2 M final concentration in 50 mM Ammonium Bicarbonate solution (ABS), followed by reduction of cysteines with 1 mM DTT (45 min; 56°C), alkylation [10 mM Iodoacetamide (IAA); 45 min; 22°C; dark] and digestion (0.015 μg Trypsin per 1.5 μg sample protein) (Trypsin Gold, Promega, United States; ratio trypsin/protein 1/100; overnight; 37°C). Digested peptide solutions, were acidified with trifluoroacetic acid (TFA) to pH < 2, desalted using STAGE tips (Rappsilber et al., 2007; Tsolis and Economou, 2017), and stored lyophilized at −20°C, until the MS analysis.

Lyophilized peptide samples were re-suspended in an aqueous solution containing 0.1% v/v formic acid (FA) and 5% v/v acetonitrile (ACN) and analyzed using nano-Reverse Phase LC coupled to a Q Exactive^TM^ Hybrid Quadrupole - Orbitrap mass spectrometer through a nanoelectrospray ion source (Thermo Scientific, Germany). Peptides were initially separated using a Dionex UltiMate 3000 UHPLC system on an EasySpray C18 column (Thermo Scientific, Germany, OD 360 μm, ID 50 μm, 15 cm length, C18 resin, 2 μm bead size) at a flow rate of 300 nL min^−1^. The LC mobile phase consisted of two different buffer solutions, an aqueous solution containing 0.1% v/v FA (Buffer A) and an aqueous solution containing 0.08% v/v FA and 80% v/v ACN (Buffer B). A 60 min multi-step gradient was used from Buffer A to Buffer B as follows [0–3 min constant (96:4), 3–15 min (90:10); 15–35 min (65:35); 35–40 min (35:65); 40–41 min (5:95); 41-50 min (5:95); 50–51 min (95:5); 51–60 min (95:5)].

The separated peptides were analyzed in the Orbitrap QE operated in positive ion mode (nanospray voltage 1.5 kV, source temperature 250°C). The instrument was operated in data-dependent acquisition (DDA) mode with a survey MS scan at a resolution of 70,000 FWHM for the mass range of m/z 400-1600 for precursor ions, followed by MS/MS scans of the top 10 most intense peaks with +2, +3, and +4 charged ions above a threshold ion count of 16,000 at 35,000 resolution. MS/MS was performed using normalized collision energy of 25% with an isolation window of 3.0 m/z, an apex trigger 5–15 s and a dynamic exclusion of 10 s. Data were acquired with Xcalibur 2.2 software (Thermo Scientific, Germany).

Raw MS files were analyzed by the MaxQuant v1.5.3.3 proteomics software package (Cox and Mann, 2008). MS/MS spectra were searched by the Andromeda search engine against the *S. lividans* TK24 proteome in Uniprot (taxonomy: 457428, originally published May, 2016 and updated May 2018, currently containing 7,505 protein entries; and common contaminants (e.g., trypsin, keratins) (Ruckert et al., 2015; Busche et al., 2018). Enzyme specificity was set to trypsin and a maximum of two missed cleavages were allowed. Dynamic (methionine oxidation and N-terminal acetylation) and fixed (*S*-carbamidomethylation of cysteinyl residues) modifications were selected. Precursor and MS/MS mass tolerance was set to 20 ppm for the first search (for the identification of the maximum number of peptides for mass and retention time calibration) and 4.5 ppm for the main search (for the refinement of the identifications). Protein and peptide false discovery rate (FDR) were set to 1%. FDR was calculated based on the number of spectra matched to peptides of a random proteome database (reversed sequence database) in relation to the number of spectra matching to the reference proteome. Peptide features were aligned between different runs and masses were matched (“match between runs” feature), with a match time window of 3 min and a mass alignment window of 20 min. Protein quantification was performed using the iBAQ algorithm (Schwanhausser et al., 2011) through MaxQuant software. Differentially abundant proteins were selected using the *t*-test and by comparing the fold difference of average protein intensities between the samples. *P*-values were further corrected for multiple hypothesis testing error using the Benjamini–Hochberg method (Benjamini and Hochberg, 1995). Thresholds for the analysis were set to adjusted *p*-value < 0.05 and fold difference >2. Functional characterization of proteomics results was performed after filtering the dataset only to secreted proteins, excluding cytoplasmic contamination, using proteome annotation as described in the SToPSdb (Tsolis et al., 2018). The percent of differentially abundant proteins that match a specific term over the total differentially abundant proteins for each condition was plotted. Keywords derived after manual curation of the proteome.

## Results

### Construction of an Arranged BAC Library of the *S. lividans* TK24 Chromosome

**FIGURE 1:**
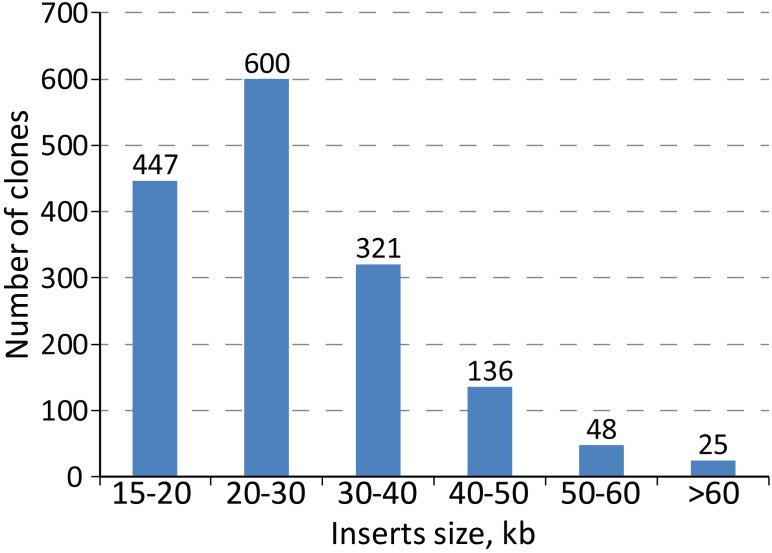
*Streptomyces lividans* TK24 BAC library inserts size distribution.

To facilitate genomic manipulation of *S. lividans* TK24, we constructed a BAC library of its chromosome. The library is based on the pSMART-Gus BAC vector (Myronovskyi et al., 2014). The presence of the *gusA* reporter gene within the vector backbone allows simple and efficient selection of double crossover events as it was demonstrated for *S. albus* (Myronovskyi et al., 2014). The resistance gene of the vector is compatible with the commonly used λ-Red recombination *Streptomyces* resistance cassettes, including both the REDIRECT and IMES systems (Gust et al., 2004; Myronovskyi et al., 2014). Due to the presence of a DNA phosphorothioation system in *S. lividans*, all library preparation steps were performed in solutions in which the Tris-HCl was replaced by HEPES (Ray et al., 1995). The genomic DNA was sheared rather than partially digested enzymatically since this provided a greater coverage of genomic fragments of large size. As a result, 1889 pSMART-Gus clones containing inserts of the *S. lividans* TK24 chromosome were individually isolated and end-sequenced. 3185 of the obtained sequences (from 1592 clones) were successfully mapped to the *S. lividans* TK24 genome. Of these, 1478 clones were correctly mapped with the end reads being inwardly oriented and paired. Of the other remaining 114 BAC clones, 38 had outwardly oriented end-reads, 48 were paired on the distances longer than 200 kbp and 29 were not paired. Manual curation of the reads resulted in 1577 clones correctly mapped to the genome (Supplementary Table 2). The average size of the inserts in the library is 27.8 kb with the smallest being 15,005 bp and largest of 86 kbp (see insert size distribution in Figure 1). The 1577 clones cover 96% of the *S. lividans* TK24 chromosome with as few as 263 out of the 7360 CDSs (3.6%) not being included in the library (Supplementary Table 3). The total length of gaps is 324.7 kbp with the largest gap of 44 kbp located at the right end of the chromosome thus excluding the predicted terpene gene cluster from the library. The positions of BAC clones on the chromosome of *S. lividans* TK24 are listed in Supplementary Table 2.

### Genomics and Transcriptomics Analysis of Sigma Factors in *S. lividans* TK24

To initiate our study of chromosomal sigma factor encoding genes of *S. lividans* TK24, we first used bioinformatics prediction and comparison with the genome of *S. coelicolor* M145. We thus determined the full compendium of sigma factors in *S. lividans* TK24. *S. lividans* TK24 is predicted to contain 63 sigma factor genes (Supplementary Table 4). However, manual curation decreased this number to 62 as both *SLIV_36640* and its ortholog from *S. coelicolor SCO0255* are coding rather for a transcriptional factor of the Fis-family. Two genes encoding *S. coelicolor* ECF sigma factors *SCO0037* and *SCO3450* are absent from the *S. lividans* TK24 genome. We also identified 19 homologues of the characterized *S. coelicolor* M145 anti-sigma factor genes being present in *S. lividans* TK24 and located in the corresponding operons as in *S. coelicolor* M145 (data not shown).

**FIGURE 2:**
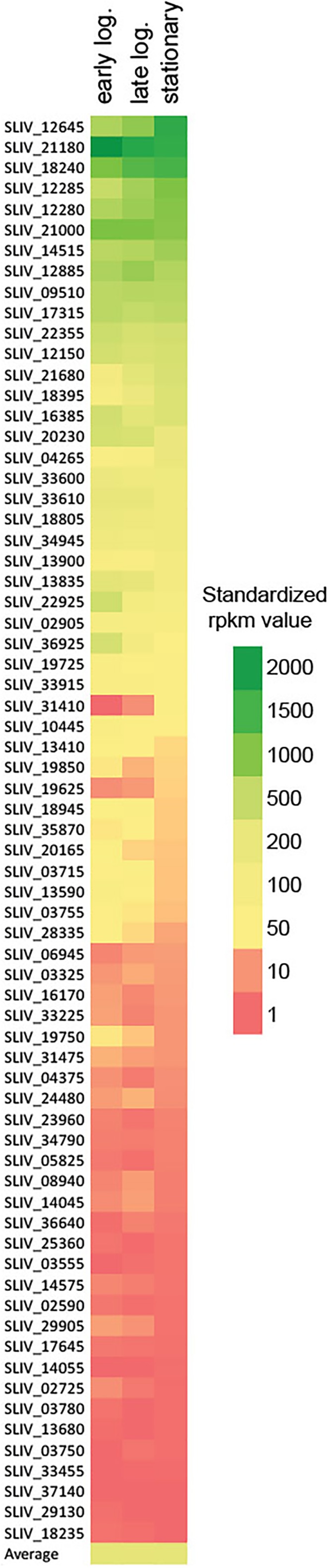
Heat-map of transcription levels of sigma factor coding genes in *S. lividans* TK24 culture at earlier and late logarithmic and stationary phases of growth. The average rpkm is shown to estimate the overall transcriptional activity of the genome under tested conditions.

To estimate the physiological importance of all individual sigma factor genes, we analyzed their transcription by RNAseq at three different time points (that correspond to early and late logarithmic, and stationary phases of growth) of *S. lividans* TK24 culture incubated in minimal medium in mini-bioreactors (Busche et al., 2018). Under these conditions the average level of transcription of the *S. lividans* genome was in the range from 228 to 240 rpkm (reads per kilobase million) with the highest expressed gene *SLIV_05305*, encoding a predicted membrane protein of unknown function (average expression level 100,741 rpkm) (Tsolis et al., 2018). This analysis revealed that out of 62 sigma factor-encoding genes 13 were transcribed at high and 22 at moderate levels (Figure 2 and Supplementary Table 4). Surprisingly, the morphological development regulators *bldN* (*SLIV_21180*) and *sigN* (*SLIV_18240*) were among the most expressed sigma factor genes surpassing the principal sigma factor *hrdB*, despite the fact that *S. lividans* is not sporulating in submerged culture. Also, in 8 out of these 35 genes transcription was induced toward the late phases of growth, while 14 were constantly transcribed throughout the growth curve and 13 showed decrease in transcription levels. 21 genes, including the *hrdC* (*SLIV_33455*), were transcribed at a very low level at all time points, thereby indicating their dispensability for the strain under the growth conditions tested.

### Construction of a Library of Sigma Factor Gene Deletion Strains in *S. lividans* TK24

Using the available BAC library, we inactivated 10 genes encoding ECF sigma factors and 6 genes coding for regulatory proteins (Supplementary Table 5). Six of the chosen genes encode for *S. coelicolor* sigma factor orthologs involved in morphological differentiation [WhiG (Chater et al., 1989), SigH (Viollier et al., 2003), SigT (Mao et al., 2009), and SigU (Gehring et al., 2001)] and stress response [SigE (Paget et al., 1999) and SigR with its anti-sigma factor RsrA (Paget et al., 1998)]. As expected, the *whiG* (*SLIV_10445*), controlling sporulation, was poorly expressed in the liquid media (Figure 2 and Supplementary Table 4). At the same time the *sigH* (*SLIV_12150*), *sigE* (*SLIV_21000*), and *sigT* (*SLIV_18805*) genes were transcribed at a high level uniformly during all periods of growth. For *sigR* (*SLIV_12285*) and its anti-sigma factor *rsrA* (*SLIV_12280*) transcription increased toward the stationary phase of growth. This might be directly related to the aging of the culture and the accumulation of reactive oxygen species. On the other hand, expression of *sigU* (*SLIV_22925*) decreased during the transition from the earlier to late log and stationary phases of growth. *SLIV_12645* (*SCO5147* ortholog, designated as *sigR1*) is one of the most highly transcribed ECF sigma factor genes in *S. lividans* TK24 and its product is phylogenetically related to SigE of *Mycobacterium tuberculosis* (Kim et al., 2012). The level of expression of *SLIV_12645* increased during growth reaching a maximum in late stationary phase. Two genes, *SLIV_16170* and *SLIV_33225*, are poorly expressed under the growth conditions tested. The functions of these sigma factors in *S. lividans* as well as their homologues in *S. coelicolor* are not known. *SLIV_14515* (*SCO4769* ortholog) codes for the ShbA sigma factor (SigD) and is highly and uniformly transcribed during the entire cultivation. Lastly, *SLIV_19850* and its *S. coelicolor* ortholog *SCO3690* codes for proteins similar to RsbQ, that controls expression of the general stress sigma factor *sigB* in *Bacillus subtilis* (Brody et al., 2001).

In addition, we selected four genes coding for the GntR family of transcription factors. The genome of *S. lividans* contains 31 genes annotated as “GntR-type” transcription factors (Ruckert et al., 2015). We have chosen two of these genes with a high level of transcription (Supplementary Table 4). The ortholog of one of them, *SLIV_17315*, was proposed to regulate xylan degradation in *S. coelicolor* (Sevillano et al., 2016), and the second (*SLIV_12885*) has a high degree of similarity to the *mngR* gene of *E. coli*, controlling 2-*O*-alpha-mannosyl-D-glycerate transport and catabolism (Sampaio et al., 2004). Both genes belong to the AdpA regulon, involved in morphological differentiation and secondary metabolism (Guyet et al., 2014). *SLIV_12885* is located in close proximity to the actinorhodin biosynthesis gene cluster. Two other genes, *SLIV_18945* and *SLIV_04375*, are transcribed at moderate and low levels, respectively. While the function of *SLIV_18945* is not known, the ortholog of *SLIV_04375* in *S. coelicolor* (*SCO6974*) is involved in regulation of myo-inositol catabolism (Yu et al., 2015).

**FIGURE 3:**
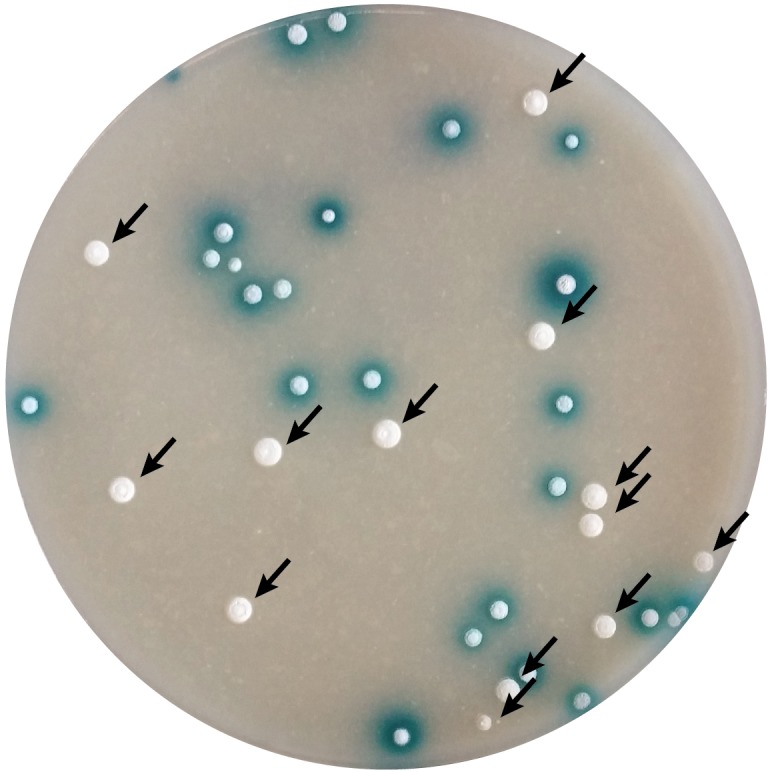
Selection of secondary cross-over mutants of *S. lividans* with the use of *gusA*-based BAC clones. Plates after conjugation were overlaid with the X-gluc solution. Blue colonies carry the single cross-over inserted recombinant BAC clone; white colonies originated from secondary cross-over event eliminating the vector backbone and thus resulting in replacement of target gene with the antibiotic resistance cassette.

To delete the selected genes the recombinant BAC clones were generated with the λ-Red recombination approach using IMES antibiotic resistance cassettes (Supplementary Table 5) (Myronovskyi et al., 2014). The mutated BACs were introduced into *S. lividans* TK24 and double cross-over strains were identified by screening for the loss of β-glucuronidase activity (Figure 3). In all cases, the double cross-over mutants were obtained directly after conjugation with an efficiency varying between experiments and solely dependent on the size of the insert of the BAC clone used. With larger insert sizes, the frequency of secondary recombination events was higher, however, the efficiency of DNA transfer decreased. On average, the secondary cross-over clones comprised 20-40% of the transconjugant populations. Fifteen mutants were obtained in 3 weeks, starting with BAC clone isolation and modification and ending with the verification of the mutant genotypes. However, we were not able to delete the *SLIV_14515* from the chromosome of *S. lividans* TK24 despite the high frequency of transconjugants with the single cross-over. This makes us hypothesize that the deficiency in the ShbA homolog is likely lethal to *S. lividans* TK24.

*Streptomyces lividans* TK24 is often used as a heterologous host for the production of secondary metabolites. Removing endogenous gene clusters is one of the major ways to improve the properties of such a “chassis” strain. To test the utility of the λ-Red-mediated targeting approach to delete large DNA fragments from the chromosome of *S. lividans* TK24 we replaced the core region of the undecylprodigiosin gene cluster (*SLIV_09100 – 09185*) by the apramycin resistance cassette. Furthermore, we successfully removed the resistance marker in the resulting strain, by expressing the integrase gene of the øC31 actinophage (Myronovskyi et al., 2014). In total, 26.5 kbp of *S. lividans* TK24 chromosome was deleted in a single step procedure.

### Phenotyping the Sigma-Factor Gene Deletion Mutants Toward Growth Related Parameters

**FIGURE 4:**
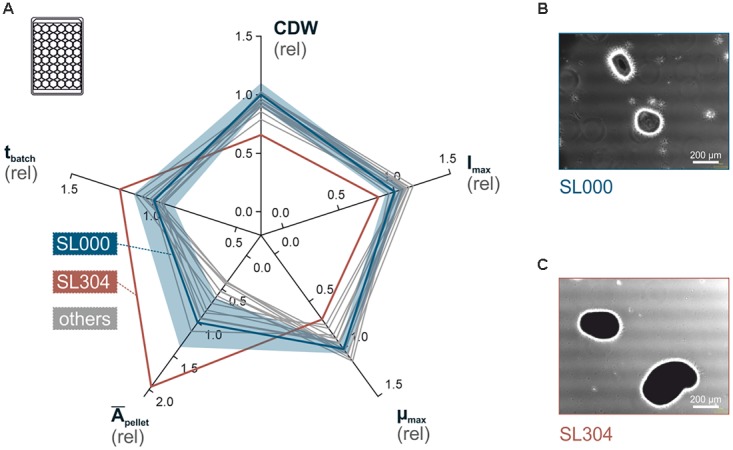
Phenotyping of *S. lividans* deletion mutants, by microscale cultivation. **(A)** Radar plot showing phenotypes of all deletion mutants relative to the wild-type (solid blue line) covering the parameters (clockwise, from top) cell dry weight (CDW), maximum scattered light intensity (I_max_), maximum specific growth rate (μ_max_), average pellet size (*Ā*_pellet_) and cultivation duration (t_batch_). For the wild-type strain TK24 (the relative standard deviation is provided (semi-transparent blue area). The complete data set with all standard deviations can be found in Supplementary Table 2). **(B,C)** Representative light-microphotographs of the *S. lividans* TK24 (SL000) **(B)** and the TK24Δ12645 mutant (SL304) **(C)**, which exposes the most prominent phenotype (white bar: 200 μm).

In a first phenotyping approach, mutant strains were characterized toward their growth performance, in comparison to the wild-type strain. As filamentous organisms are especially sensitive to varying cultivation conditions (Siebenberg et al., 2010; Fieser et al., 2015), it was a basic requirement to ensure highly comparable growth parameters and a well-controlled cultivation environment for all strains. Therefore, we applied a recently developed phenotyping workflow, which utilizes micro-bioreactor cultivation, subsequent to a two-step stirred flask pre-culture procedure (Supplementary Figure 1) (Koepff et al., 2017). This pipeline enabled the characterization of all strains in triplicates within an acceptable workload toward maximum growth rate (μ_*max*_), cell dry weight concentration (CDW), maximum scattered light intensity (I_*max*_), batch time duration (t_*batch*_) and average projected pellet area (*Ā*_*pellet*_) (Figure 4A and Supplementary Table 6).

Most mutants did not show much of a difference in comparison to the wild-type *S. lividans* TK24. The deletion of selected genes did not have a significant influence on measured growth parameters. The *Ā*_*pellet*_ parameter, derived from light-microscopy and subsequent automated image analysis, demonstrated the largest data scatter between all investigated strains. However, even *S. lividans* TK24 exhibits a broad pellet size distribution (standard deviation of ±36%).

The only strain that demonstrated clear differences in growth when compared to *S. lividans* TK24 was TK24Δ12645 (Figure 4). It showed significant reduction of biomass accumulation leading to 15% lower I_*max*_ and 34% decreased CDW. Furthermore, the maximum growth rate μ_*max*_ was reduced by 31%, while t_*batch*_ was increased by 21%. Finally, the culture average pellet size (*Ā*_*pellet*_) of TK24Δ12645 was almost double to that of the wild-type *S. lividans* TK24.

### Effect of Mutations on Morphology and Secondary Metabolism

**FIGURE 5:**
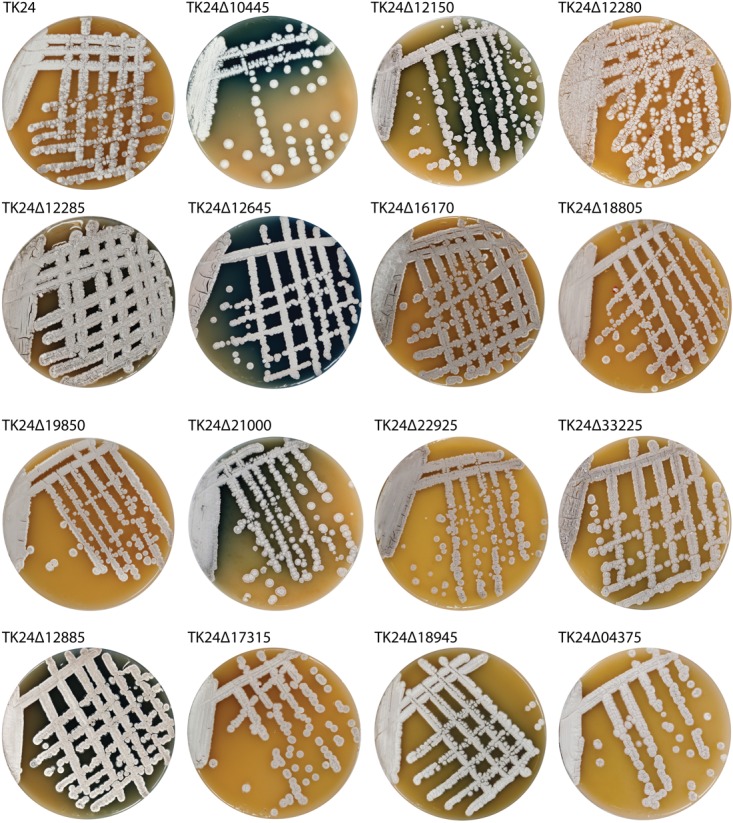
*S. lividans* TK24 and its mutant’s phenotype while grown on rich solid MS medium. Blue coloration corresponds to accumulation of actinorhodin.

When grown on rich solid medium, most of the strains do not display much difference when compared to *S. lividans* TK24 (Figure 5). The only exception is TK24Δ10445. As expected, deletion of the *whiG* ortholog caused a lack of spore pigment resulting in white rather than grey colonies. Also, several mutants affected secondary metabolites production. *S. lividans* TK24 does not accumulate pigmented compounds during growth on MS or minimal media. Under these conditions seven strains, TK24Δ10445, TK24Δ12150, TK24Δ12285, TK24Δ12645, TK24Δ21000, TK24Δ12885 and TK24Δ18945, are producing actinorhodin (Figure 5). Interestingly, TK24Δ12885, depending on the density of its colonies, accumulates different pigmented compounds: when grown at lower density, actinorhodin is accumulated; in lawn culture the strain is producing undecylprodigiosin (Supplementary Figure 2). TK24Δ10445 produces undecylprodigiosin when grown on minimal media (Supplementary Figure 3). TK24Δ12645 exerted the most dramatic influence on secondary metabolism of *S. lividans*. TK24Δ12645 accumulated large quantities of actinorhodin on MS, TSB and solid minimal media (Figure 5 and Supplementary Figures 2–4). Furthermore, the coelimycin P1 gene cluster, considered to be silent in *S. coelicolor* and *S. livindans*, is ectopically upregulated in TK24Δ12645 strain, resulting in accumulation of a yellow pigment between 24 and 48 h of growth (Supplementary Figure 5) (Gomez-Escribano et al., 2012). Similar to *S. coelicolor*, the coelimycin is cleared from the media after 60 h of cultivation (Gottelt et al., 2010). TK24Δ12645 is also accumulating undecylprodigiosin in liquid minimal medium and actinorhodin in liquid TSB, NL19 and SG media (Supplementary Figures 6, 7).

### Effect of Sigma Factor Gene Deletions on Stress Tolerance

Several of the deleted sigma factor genes are known to be involved in oxidative, cell-wall and disulfide stress responses in *S. coelicolor* (Paget et al., 2002). We have tested the sensitivity of *S. lividans* TK24 and its derivative mutants to diamide, vancomycin and hydrogen peroxide by the disk diffusion assay. Diamide, a thiol-specific oxidizing agent, causes the formation of disulfides in low-molecular-weight thiols and in proteins, thereby affecting protein folding (Kosower and Kosower, 1995). Vancomycin, a cell wall specific antibiotic, was used to probe the cell wall stress tolerance (Bugg et al., 1991; Hesketh et al., 2011); and hydrogen peroxide was used as a general oxidative stress inducer (Juven and Pierson, 1996). As expected, TK24Δ12285, coding for a SigR ortholog, caused the decrease in diamide tolerance (Table 1) (Paget et al., 1998). A similar effect was observed in the case of TK24Δ10445, TK24Δ 18805, and TK24Δ19850 with the more dramatic effect observed on minimal media. Reversely, inactivation of the SigR-specific anti-sigma factor gene *SLIV_12280* led to a slight increase in diamide resistance. Five other strains TK24Δ21000, TK24Δ12150, TK24Δ12645, TK24Δ16170, and TK24Δ33225 demonstrated different degrees of increased tolerance to sulfate-oxidizing stress. Among them, the most dramatic difference in resistance was observed in TK24Δ12645 with the growth inhibition zone completely overgrown after 120 h of cultivation (Supplementary Figure 8). On the other hand, no significant difference in tolerance to hydrogen peroxide between the wild-type and the generated deletion mutants was observed (data not shown). However, several strains accumulated actinorhodin around the hydrogen peroxide discs, including TK24Δ10445, TK24Δ12285, TK24Δ12885, and TK24Δ18805 (Supplementary Figure 9). *SLIV_21000* encodes the ortholog of *S. coelicolor* SigE, involved in sensing and response to cell-wall stress (Hesketh et al., 2011). TK24Δ21000 was the only strain with significantly increased sensitivity to vancomycin when compared to the wild-type TK24 (Supplementary Figure 10).

**Table 1:** Zones of growth inhibition of *S. lividans* mutant strains by diamide determined by disk diffusion assay.

Stain	*S. coelicolor* ortholog		MS, mm 10 μM	TSB, mm 10 μM	Min.M, mm 10 μM
*S. lividans* TK24			33 ± 1.4	34 ± 0.4	33 ± 2.8
Δ 10445	SCO5621	WhiG	34 ± 0.9	34 ± 2.3	46 ± 3.3
Δ 12150	SCO5243	SigH	32 ± 0.9	27 ± 0.9	29 ± 1.8
Δ 12280	SCO5217	RsrA	24 ± 3.7	30 ± 0.4	28 ± 1.7
Δ 12285	SCO5216	SigR	36 ± 0.4	39 ± 0.4	45 ± 3.3
Δ 12645	SCO5147	SigR1	19 ± 0.9	19 ± 2.7	18 ± 2.7
Δ 18805	SCO3892	SigT	34 ± 1.4	36 ± 0.9	48 ± 3.3
Δ 19850	SCO3690	RsbQ	32 ± 0.4	34 ± 0.4	40 ± 1.4
Δ 16170	SCO4452	SigL1	23 ± 1.4	26 ± 2.3	19 ± 2.3
Δ 21000	SCO3356	SigE	23 ± 0.4	21 ± 2.3	27 ± 2.3
Δ 22925	SCO2954	SigU	33 ± 0.9	31 ± 2.3	32 ± 3.7
Δ 33225	SCO0942	SigL	22 ± 0.4	21 ± 2.7	29 ± 0.9
Δ 12885	SCO5100	GntR1	30 ± 1.8	35 ± 0.9	34 ± 2.7
Δ 17315	SCO4215	GntR2	32 ± 0.4	34 ± 0.4	34 ± 3.7
Δ 18945	SCO3864	GntR3	33 ± 0.4	34 ± 0.4	34 ± 3.3
Δ 04375	SCO6974	GntR4	30 ± 2.3	34 ± 0.4	31 ± 2.3

### Effect of Sigma Factor Gene Deletions on the Secretome

**FIGURE 6:**
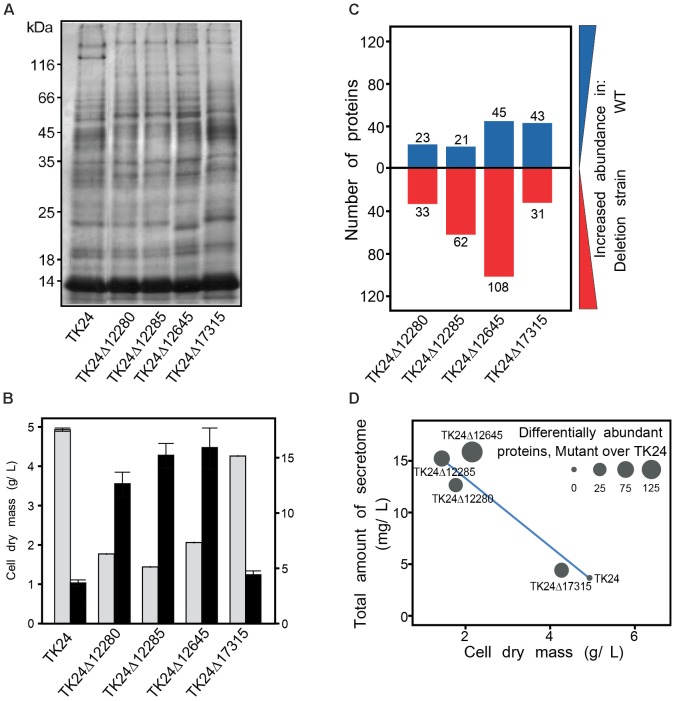
Secretome analysis of sigma factor gene deletion mutants. **(A)** Secretome profile of *S. lividans* TK24 and derivatives with sigma factor gene deletions. Polypeptides were resolved by SDS-PAGE and silver stained, loading equal amount of secretome polypeptides (3 μg). **(B)** Cell dry weight (g/L) (light gray) and amount of total secreted proteins (mg/L) (black) for the strains used in the proteomic analysis. **(C)** Number of differentially abundant secreted proteins between each mutant against the wild type. Proteins with increased abundance in *S. lividans* TK24 are shown in blue and proteins more abundant in deletion strains are colored red. Samples were loaded for proteomic analysis normalized to the same amount of cell biomass. **(D)** Correlation between dry cell weight, amount of total secreted proteins and number of differentially abundant proteins over the WT. (*n* = 4–7).

*Streptomyces lividans* is an established host for secreted protein production and many of the sigma factor genes deleted here are expected to affect extracellular functions. Therefore, we were interested to test the influence of selected deletions of sigma factor-encoding genes on the secreted proteome of *S. lividans* TK24. For this, we examined the secretomes of 4 mutant strains in comparison to the parental *S. lividans* TK24 (Figure 6). Equal amounts of total secretome polypeptides were first analyzed by SDS-PAGE and silver staining (Figure 6A). The patterns of the various derivatives looked similar overall at this level of analysis except for TK24Δ12645 that seems to secrete several new polypeptides and had lost others (Figure 6A). However, upon loading of secretome material derived from the same volume of culture, TK24Δ12645 was also seen to be a profuse secretor of polypeptides (not shown). As seen in other studies (Tsolis et al., 2018), there appears to be a good correlation between suppressed growth and improved secretion as seen by comparison of the total secretome expressed per unit cell biomass (Figure 6B). The same polypeptides were also analyzed by label-free nanoLC-MS/MS, the identity of the polypeptides determined and their amounts quantified (Supplementary Table 7). The abundance of proteins in the secretome of the deletion derivative strains was compared to that of the wild-type (Figures 6C,D). In all cases, several polypeptides, representing 10–20% of the secretome, were identified at abundances different to those of the wild-type.

### Secretome Analysis of *S. lividans* TK24Δ12645

**FIGURE 7:**
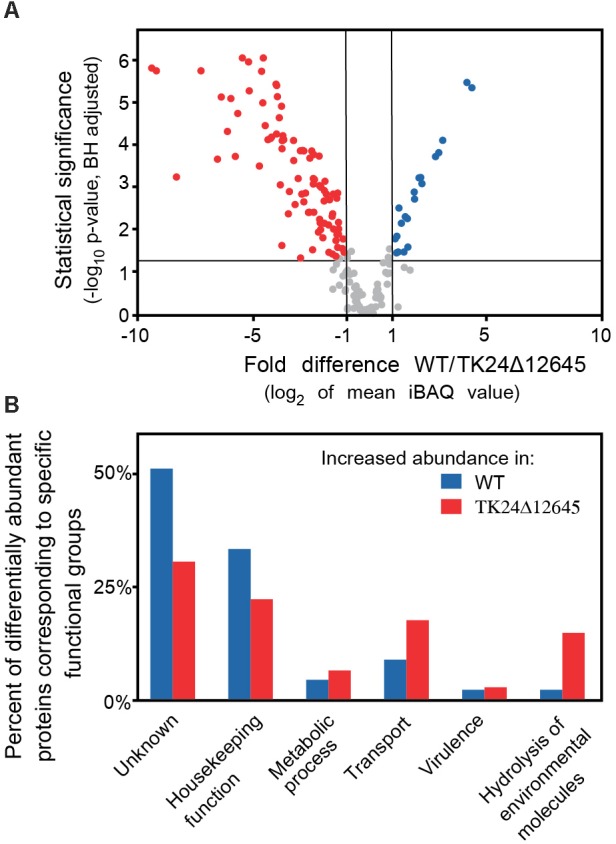
Comparative secretomics of *S. lividans* TK24 and TK24Δ12645. **(A)** Volcano-plot showing the differentially abundant proteins between *S. lividans* TK24 and TK24Δ12645. Each dot represents one protein. On the *x* axis is plotted the fold difference (in log_2_ scale) of the mean protein abundance in the *S. lividans* TK24 over that in the TK24Δ12645, and on the *y* axis the *p*-value derived from a *t*-test between the two strains (–log_10_, adjusted by Benjamini–Hochberg method). With blue are colored proteins more abundant in the TK24 and in red, the significantly more abundant proteins in TK24Δ12645. **(B)** Functional classification of differentially abundant proteins based on their biological function. The ratio of the abundant proteins corresponding to a specific functional category over the number of over-represented proteins in the specific strain is plotted.

Finally, we analyzed in detail the secretome of *S. lividans* TK24Δ12645 and compared it to that of the wild-type TK24. The abundance of 153 secreted proteins is statistically different in the two strains, reflecting proteins that are seen at both lower and higher levels in the mutant strain (Figure 7A and Supplementary Table 7). Proteins affected include a putative protein foldase, a subtilisin-family peptidase and a putative lipoprotein localization LolA/LolB/LppX-like protein that are synthesized/secreted > 5 times less than in *S. lividans* TK24 and a phospolipase, a secreted peptidase of S1 family and a neutral zinc metalloprotease that are secreted > 5-fold more. Overall, the affected proteins fall in 6 main functional classes (Figure 7B). This is suggestive of an extensive regulatory role of SigR1 (SLIV_12645) in *S. lividans.*

## Discussion

*Streptomyces coelicolor* and *S. lividans* are two closely related species that are used for decades as models for basic genetic, physiological, biochemical and morphological research of actinobacteria. The extensive exploitation of *S. coelicolor* promoted development of genetic tools for this strain, including protoplast transformation and fusion (Noack et al., 1984), intergeneric conjugation (Mazodier et al., 1989), λ-Red mediated PCR targeting (requires genomic library construction) (Gust et al., 2004) and finally CRISPR/Cas genome editing technique (Tong et al., 2015). All these tools with some conditions can be applied to other streptomycetes. At least for now the λ-Red mediated PCR targeting remains the most efficient tool for genomic manipulations in streptomycetes. The major bottleneck of the λ-Red approach is the need for an arranged genomic library of the strain of interest and marker re-utilization. Genomic libraries are available for several important *Streptomyces* strains, including *S. coelicolor* M145 (Redenbach et al., 1996; Fernandez-Martinez et al., 2011), *S. lividans* 66 (Zhou et al., 2004), *S. albus* J1074 (Myronovskyi et al., 2014), but not *S. lividans* TK24. Despite being used for a long time as a model for basic research, an excellent host for secondary metabolites gene expression and protein production, the gene deletions in this strain are mainly done with the conventional approaches (i.e., using suicide or temperature-sensitive plasmids) that are time consuming and require conventional cloning steps. To facilitate further development of *S. lividans* TK24 as a chassis strain for biotechnological applications and to enable fast and precise genome engineering we have constructed a genomic library built on the BAC-vector with the *gusA* reporter gene inserted in the backbone that allows positive selection of colonies with secondary cross-over, facilitating direct identification of mutant colonies (Myronovskyi et al., 2011). The relatively small insert size in the library can be attributed to difficulties in isolating high quality and integrity chromosomal DNA from *S. lividans*. This was due to the presence of a site specific DNA phosphorothioate modification system (Zhou et al., 1988; Ray et al., 1995). This unusual DNA modification, replaces the oxygen atom in the phosphate group of the DNA backbone with sulfur, can be found in many bacteria, including 35 streptomycetes (Tong et al., 2018). It may play a role similar to restriction-modification systems; however, recent findings suggest its broader involvement in bacterial physiology by protecting DNA from oxidative stress and influencing overall transcriptional activity (Dai et al., 2016; Tong et al., 2018). The DNA phosphorothioate modification system should be considered during DNA manipulations, especially during genomic library construction.

The BAC-based gene deletion approach allowed us to delete 15 genes coding for sigma factors, anti-sigma factors and regulatory proteins as well as a major part of undecylprodigiosin biosynthesis gene cluster. The entire procedure starting with BAC isolation and modification and ending with verification of mutant strain genotypes took less than 3 weeks. This is much faster than conventional cloning-based approaches and much safer (in terms of unspecific mutations) than current versions of the CRISPR/Cas technique (Huang et al., 2015). The major advantage of this approach is the possibility of direct selection of secondary cross-over mutants right after conjugation due to large homologous arms and the *gusA* reporter gene. Combined with the IMES marker system, the BAC-library also allows for multiple gene deletion in the same genetic background making it an indispensable tool for *S. lividans* strain engineering until the more precise and accurate CRISP/Cas system becomes available.

Transcriptome analysis of *S. lividans* TK24 grown in liquid culture revealed a group of sigma factor genes that are actively transcribed during culture growth, including one encoding the alternative and ECF sigma factors. The observed transcription level of *hrdB* gene (*SLIV_09510*), encoding a principal sigma factor in *S. coelicolor* (Buttner et al., 1990), is even during all stages of growth of the culture. Several sigma factor genes, including *SLIV_12285* (SigR), *SLIV_12645* (SigR1), *SLIV_18240* (SigN), *SLIV_14515* (SigD, ShbA), *SLIV_21680* (HrdD), *SLIV_18395* and *SLIV_31410* are strongly upregulated, while that of others downregulated in stationary phase of growth. The latter include *SLIV_21000* (SigE), *SLIV_22925* (SigU), *SLIV_36925* (LitS), and *SLIV_20230*. These differences may be indicative of an involvement of these two distinct groups of sigma factors in transcription of genes required either for rapid vegetative growth or persistence under limiting conditions. As such, SigE is involved in cell wall stress sensing and response and thus is in high demand during active phases of cell division and growth, but not at stationary phase (Paget et al., 1999). Conversely, sigma factor genes overexpressed in late phases of growth most probably are involved in conservation and persistence of the culture, mainly by increasing tolerance to low nutrition and high stress conditions. Thus, it is not surprising that transcription of *sigR* (*SLIV_12285*) is significantly increased in stationary phase of growth, presumably to ensure the effective expression of the oxidative stress response regulon (Paget et al., 1998; Kim et al., 2012). Surprisingly, among genes induced during late growth was *sigN* (*SLIV_18240*), involved in regulation of morphological differentiation (Dalton et al., 2007) (Supplementary Table 4). Furthermore, the most highly transcribed sigma factor gene under the conditions tested was *bldN* (*SLIV_21180*). Transcription levels of *bldG* (*SLIV_20540*), encoding for the anti-sigma factor RsbV, and of *bldH* (*SLIV_23715*) that together activate bldN gene transcription (Bibb et al., 2000), are also significantly elevated in liquid culture (average 3956 and 359 rpkm, respectively) (Supplementary Table 8). At the same time, the negative regulator of bldN transcription, *bldD* (*SLIV_30325*) is also expressed at a high level during all periods of cultivation (average 927 rpkm) (den Hengst et al., 2010); as well as the major members of the BldN regulon: *bldM* (*SLIV_14520*) and genes encoding chaplins and rodlins (Bibb et al., 2012). All these gene products are involved in aerial mycelium formation and sporulation. A similar trend in the transcription of developmental genes was observed in *S. coelicolor* cultures grown in liquid minimal medium, despite the fact that neither *S. coelicolor* nor *S. lividans* form spores in submerged cultures (Nieselt et al., 2010; Yague et al., 2014). It is proposed that *S. coelicolor* in liquid culture is preparing for spore formation by inducing expression of genes responsible for formation of the hydrophobic cover of mycelia; however, the differences in growth conditions are blocking the next steps of the developmental program. One of the possible genetic reasons for this phenomenon could be permanent high level expression of *bldD* or *sigU* (*SLIV_22925*), known to delay sporulation (Gehring et al., 2001).

Of the sixteen initially targeted genes only one, *sigD* (or *shbA*, *SLIV_14515*; ortholog *SCO4769*), could not be deleted, suggesting that it may be essential for *S. lividans*. Its transcription level increased toward the stationary phase, probably indicating its importance in late phase cultures. Its homologue in *S. griseus* drives transcription of the principal sigma factor gene (Otani et al., 2013) and its deletion caused significant alterations in growth, morphology, and in the expression levels of numerous housekeeping genes.

Deletion of most of the remaining targeted genes did not cause any prominent phenotypes at first glance, probably indicating their narrow specialization. As expected, deletion of *whiG* (*SLIV_10445*) caused defects in sporulation (Chater, 1972); deletion of *sigR* and *sigE* leads to increased sensitivity to diamide and vancomycin, respectively, pointing to their function in regulation of thiol-oxidative and cell-wall stresses response (Paget et al., 1998; Hesketh et al., 2011). The most dramatic changes, however, were observed in the case of TK24Δ12645. *SLIV_12645* encodes the ECF sigma factor SigR1; its transcription is strongly upregulated in stationary phase cultures (Supplementary Table 4). The ortholog of *SLIV_12645* (*SCO5147*) was thought to be essential for viability in *S. coelicolor* (Kang et al., 2007). However, inactivation of SCO5147 with a transposon insertion caused a slight increase in vancomycin resistance (Hesketh et al., 2011); although, we did not observe any changes in tolerance to this cell-wall specific antibiotic in TK24Δ12645, probably due to the different conditions used. Nevertheless, deletion of *SLIV_12645* caused retarded culture growth, lower biomass accumulation, affected morphology in liquid culture and accumulated different antibiotics (Figure 5 and Supplementary Figures 2–4). We anticipate that this influence on antibiotics production is a general reaction of the strain to the lack of SigR1, rather than being suggestive of a specific involvement of *SLIV_12645* in regulating secondary metabolism in *S. lividans* TK24. A similar phenomenon was observed in the case of SigE-deficient *S. coelicolor* over-producing actinorhodin (Paget et al., 1999). *sigE* mutant phenotype was suppressed by presence of Mg^2+^ ions that stabilize the cell wall structure. Thus it was proposed that overproduction of actinorhodin by the sigE mutant strain is caused by the permanent cell wall stress. Indeed we observed induction of actinorhodin production by the *S. lividans* strain lacking *SLIV_21000* gene, coding for SigE ortholog. We think that similar scenario take place also in the case of strain lacking *SLIV_12645* encoded SigR1.

More insight on the effects of deletion of *SLIV_12645* was revealed by analysis of its secreted proteome. Secreted proteins with affected levels in TK24Δ12645 fell in 6 main functional classes (Figure 7B). This is suggestive of an extensive regulatory role of SigR1 in *S. lividans*, and presumably in *S. coelicolor*, that may be exerted through a combination of mechanisms that involve transcriptional control both at the level of the individual exported protein genes and/or at the level of chaperone and secretion machinery. Little is known about which of these pathways are under SLIV_12645 control. Among the most underrepresented proteins in the secretome of TK24Δ12645 strain are proteins involved in morphological development and cell wall biosynthesis and remodeling: PBP2 (SLIV_27255), D-Ala-D-Ala-carboxypeptidase 3 (SLIV_07480), RpfD (Resuscitation-Promoting Factor, peptidoglycan cleavage, SLIV_33070), peptidoglycan amidase (SLIV_11070), NlpC/P60 subgroup amidase (SLIV_17370), endopeptidase (SLIV_04650), putative amidase (SLIV_06435), MreC (SLIV_24625) are exclusively found in the *S. lividans* TK24 secreted proteome (Gordon et al., 2008; Haiser et al., 2009; Ladwig et al., 2015; Sexton et al., 2015). Also, putative peptidoglycan endopeptidases (SLIV_09410 and SLIV_04140), three class B high molecular weight PBPs (SLIV_19035, SLIV_18345, SLIV_28340), peptidoglycan carboxypeptidase (SLIV_22865) proteins are less abundant in the TK24Δ12645 strain (Supplementary Table 7). In addition, several other proteins with functions in cell division or membrane protein homeostasis are also affected by deletion of *SLIV_12645* gene. Genes encoding the aforementioned secreted proteins may be part of a SigR1 regulon. Transcription of several gene orthologs was affected in *S. coelicolor* upon treatment with antibiotics targeting the cell wall (encoding PBPs: *SLIV_27255*, *SLIV_18345*, *SLIV_19035*, *SLIV_28340*; putative peptidoglycan amidase gene *SLIV_11070*, *rpfD SLIV_33070*) (Hesketh et al., 2011, 2015).

Proteins that are more abundant in TK24Δ12645 also fall into several groups. The largest of them includes 12 proteins involved in cell wall biogenesis and remodeling (Supplementary Table 7). The over-secretion of these proteins may be part of compensatory mechanism by which *S. lividans* responds to the lack of a group of cell-wall biosynthesis and remodeling proteins caused by the absence of SigR1. Increased accumulation of 5 proteins implemented in heat-shock, osmotic and redox stress might be a part of same compensatory response (Supplementary Table 7). Interestingly, the negative regulator of *sigE*, CseA (SLIV_20995) is also more abundant in the secreted proteome of TK24Δ12645, implying a possible direct link between SigE and SigR1. Several members of the PhoP/R regulon are overrepresented in the secreted proteome of TK24Δ12645, as well as some proteins involved in morphological development and cell division regulation, including BldKB and SapA (hydrophobic cover protein). Lastly, the abundance of secreted proteins associated with secondary metabolism is also increased in TK24Δ12645. Most of them are involved in binding and transport of metal-siderophore complexes (SLIV_02315 and SLIV_23775 (ferrioxamine uptake), SLIV_00915 (coelibactin-Zn uptake) and SLIV_35470 (CchF, coelichelin uptake); while three proteins are involved in biosynthesis of actinorhodin (SLIV_13015, ActVI-ORF3) and calcium-dependent antibiotic (CDA, SLIV_21580 and SLIV_21470) (Supplementary Table 7).

Taken together, the data point to the involvement of SigR1 (SLIV_12645) in processes of cell wall biosynthesis and re-modeling. The previous observations also suggest its *S. coelicolor* ortholog involvement together with the SigR in transcription of redox stress response genes (Kim et al., 2012). This makes us think, that SigR1 most probably acts similarly to SigE of *Mycobacterium tuberculosis* (Manganelli et al., 2001), responding to both oxidative and surface stresses and modulating respective response genes transcription. Despite its obvious importance, the function of SigR1 remains unclear and will require further investigation.

In addition to TK24Δ12645, three more of the tested mutant strains, show changes in the abundance of 10–20% of their secreted proteins, compared to that of the wild-type (Figure 6C). This suggested that the genes encoding secreted proteins or protein secretion machineries are controlled by the specific sigma factors and are sensitive to their lack and yet, given the minor effect on fitness (Figure 4A), cellular proteostasis remains robust. This implies a high degree of build-in redundancy and system robustness. Complex networks of sigma factors may cover up for the absence of each other and allows at least some transcription from alternative promoters. Another, complexity comes from the effects of anti-sigma factors: anti-sigma factors acting in the absence of their regular client sigma factors may form associations with other non-optimal client sigma factors and affect their transcriptional roles. Currently, the molecular basis of these balanced but complex networks remains unknown and will require further future scrutiny.

To summarize, we have developed an efficient and robust system for genome engineering of *S. lividans* TK24, based on λ-Red recombination and a genomic BAC library. This system will further facilitate work on *S. lividans* strain improvement as a host for either secondary metabolites or protein production and will contribute to basic research in streptomycetes physiology, morphological development, secondary metabolism, thus overall increasing our understanding of these important bacteria.

## Author Contributions

YR, LH, MM, and BB generated mutants. YA conducted deletion of *upg*-gene cluster. TB and CR performed RNAseq experiments. EG constructed genomic library. JK, AB, WW, and YR performed phenotyping and fermenter cultivation. MH and KT performed secretome characterization. JA, ÓF, JK, MO, AE, and AL planned the work and designed the experiments. YR wrote the paper with contributions from AL and AE. All authors contributed to the manuscript preparation, discussion, and editing.

## Conflict of Interest Statement

The authors declare that the research was conducted in the absence of any commercial or financial relationships that could be construed as a potential conflict of interest.

## Footnotes

1 http://www.geneious.com
