# Supplementary material for: Characterization of Sigma Factor Genes in Streptomyces lividans TK24 Using a Genomic Library-Based Approach for Multiple Gene Deletions
Source: Front Microbiol. 2018 Dec 10;9:3033. doi: 10.3389/fmicb.2018.03033 (PMC6295645; doi:10.3389/fmicb.2018.03033)
Supplement: Supplementary file 1 [file Data_Sheet_1.pdf]

Supplementary Material

Genomic library based approach for multiple gene deletion in *Streptomyces lividans* TK24: characterization of sigma factor genes.

Yuriy Rebets [1#], Konstantinos C. Tsolis [2#], Elísabet Eik Guðmundsdóttir [3#], Joachim Koepff [4,5#], Beata Wawiernia [3#], Tobias Busche [6], Arne Bleidt [4], Liliya Horbal [1], Maksym Myronovskyi [1], Yousra Ahmed [1], Wolfgang Wiechert [4], Christian Rückert [6], Mohamed B. Hamed [2,7], Bohdan Bilyk [1,8], Jozef Anné [2], Ólafur Friðjónsson [3], Jörn Kalinowski [6], Marco Oldiges [4,5], Anastassios Economou [2], Andriy Luzhetskyy [1]

[#] these authors equally contributed to this work.

*** Correspondence:** Andriy Luzhetskyy: a.luzhetskyy@mx.uni-saarland.de

Supplementary Data

Supplementary Table 1. Primers used in this work.

Supplementary Table 2. List and coordinates of BAC clones of *S. lividans* TK24 genomic BAC library (additional file).

Supplementary Table 3. Location and size of *S. lividans* TK24 chromosome regions not covered in the BAC library.

Supplementary Table 4. List of sigma factors in *S.lividans* TK24 and *S.ceolicolor* M145 and results of transcriptomics analysis (additional file).

Supplementary Table 5. List of strains generated in this study.

Supplementary Table 6. Data table for microtiter-plate strain phenotyping.

Supplementary Table 7. *S. lividans* secretome changes in sigma factor gene deletion strains determined by nanoLC-MS/MS.

Supplementary Table 8. Transcription of *S. lividans* TK24 *bldN* orthologue (*SLIV_21180*) and its regulon during cultivation in liquid minimal medium (additional file).

Supplementary Figure 1 Fitness testing of sigma factor deletion strains using microtiter-plate based screening workflow.

Supplementary Figure 2. Growth of *S. lividans* TK24 and mutants lacking *SLIV_12645* and *SLIV_12885* on MS agar plates.

Supplementary Figure 3. Growth of *S. lividans* TK24 and mutants lacking *SLIV_12645* and *SLIV_10445* on Minimal medium plates.

Supplementary Figure 4. Growth of *S. lividans* TK24 and mutant lacking *SLIV_12645* on TSB agar plate.

Supplementary Figure 5. Production of different secondary metabolites by *S. lividans* TK24Δ12645 at different time points of cultivations on MS agar plates.

Supplementary Figure 6. Accumulation of secondary metabolites by *S. lividans* TK24 and TK24Δ12645 mutant cultivated in different liquid media.

Supplementary Figure 7. Microtiter-plate cultivation of *S. lividans* TK24 and TK24Δ12645 in liquid minimal medium.

Supplementary Figure 8. Sensitivity of *S. lividans* TK24 and TK24Δ12645 mutant to 10 µM of diamide after 120 hours of incubation at 28°**.**

Supplementary Figure 9. Induction of actinorhodin production by hydrogen peroxide in *S. lividans* TK24 mutants lacking sigma factor coding genes.

Supplementary Figure 10. Vancomycin sensitivity of *S. lividans* TK24 and TK24Δ21000 mutant grown on TSB agar plates.

Supplementary Table 1. Primers used in this work. Primers for gene deletion are marked with suffix “D”, primers used for verification of mutant’s phenotype are marked with suffix “Ch”. Primers used for *upg*-gene cluster deletion are names as “Cl10_”. Sequences annealing to the IMES antibiotic resistance cassettes are highlighted in yellow.

SLIV_10445DisF	CAGATCCACATCACGGCAGAACGGCACTAGGCGACGAATGTCGACCCGGTACCGGAGTA
SLIV_10445DisR	GGTGGGGCCGGGCCCGTGCCGTAAGCCCAGGTCAGCGGCCACTACGCCCCCAACTGAGAG
SLIV_ 12150DisF	CGGGACCCGGGCCGACGTGAGGGACGGGGACGGGCCGGTTCGACCCGGTACCGGAGTA
SLIV_ 12150DisR	CACCCGCCGACCGGCGGTTACTCCTCGACGAGCAGCTTCTACTACGCCCCCAACTGAGAG
SLIV_12280DisF	TCGAACGAAGCGAAAGGCTCGGGGTCATGAGCTGCGGAGATCGACCCGGTACCGGAGTA
SLIV_12280DisR	GTGACTTCTGACCGAGTCGCGACGCTCAGGGCGGCTCAGGACTACGCCCCCAACTGAGAG
SLIV_12285DisF	TCAGCACCGACGCCGTAGGAGAGGAAGTCCGCCGCATGGGTCGACCCGGTACCGGAGTA
SLIV_12285DisR	CCGTCTCGTGCGGCTCTCCGCAGCTCATGACCCCGAGCCTACTACGCCCCCAACTGAGAG
SLIV_12645DisF	AAACCGCTTATCCGGAGCTAACGGGCGAGGTGGCTATGGTTCGACCCGGTACCGGAGTA
SLIV_12645DisR	GTCCCTCGGGCTCAGGCCGTGACCCACTCACGCGCTCGTGACTACGCCCCCAACTGAGAG
SLIV_14515DisF	AGCCACGGATCGGACGGCCGCAAGGCACCATGGACCGATGTCGACCCGGTACCGGAGTA
SLIV_14515DisR	CGTACGAGAGGGGCCGCTGCGCGGGGCGTCGTCCGGACTAACTACGCCCCCAACTGAGAG
SLIV_18805DisF	ACCAGCTGACGGCACCTACCGCAGGGAGAGGGGTGTGATGTCGACCCGGTACCGGAGTA
SLIV_18805DisR	GCCCGGTCATGTCTGTCGTCGACGACGTCACGCTCGTCCAACTACGCCCCCAACTGAGAG
SLIV_19850DisF	ACCGATGACTGACTCGACGCCTTGGAGGACGTACCGCATGTCGACCCGGTACCGGAGTA
SLIV_19850DisR	GGTTCTCGCCCTGGTCACCGGCCCCGCGCATCAATCCGCAACTACGCCCCCAACTGAGAG
SLIV_16170DisF	CGAACGTCACGGACTGACCCCGCCGATGGACGAGGCCCTGTCGACCCGGTACCGGAGTA
SLIV_16170DisR	CGCCCACGGCGACGGGAGTTGAGGCGGGCCGCCTGGCGGGACTACGCCCCCAACTGAGAG
SLIV_21000DisF	ACCACCGTCGGAGTACGGGGATCGGAAGGCGGTTGACATGTCGACCCGGTACCGGAGTA
SLIV_21000DisR	CCGACCGTCGGTCAGGCCGCGCAACGCTCCCGCTCCTCACACTACGCCCCCAACTGAGAG
SLIV_22925DisF	CGTGGCCAATGAACGTGGTGCGAGGGCCCGACATCGCATGTCGACCCGGTACCGGAGTA
SLIV_22925DisR	CCGAATCCCTGTACTCCCCCGTAGCCGCTCATCACGCCGTACTACGCCCCCAACTGAGAG
SLIV_33225DisF	CCCTGTCCCCGCACGGTCCACTGCGGTGCCATACGGCCATTCGACCCGGTACCGGAGTA
SLIV_33225DisR	AAGGCGCTTTACGGAGGCTTGACCAAAGGGTCGGTTTCACACTACGCCCCCAACTGAGAG
SLIV_12885DF	CCACGCTGTTGCTGTCTGTACAACCTGATGGGAGCGGCCAGTGTCGACCCGGTACCGGAGTA
SLIV_12885DR	GCCACGGCCCTGCTTGAGGGGCTGCCCGACGGGCTACCTGAACTACGCCCCCAACTGAGAG
SLIV_17315DF	GGCCTAAGGTGTCTAGAGAAGGAGAGGAGGCCCACGGGATTCGACCCGGTACCGGAGTA
SLIV_17315DR	TCGTGACTCAGGCCGTGGCGTGGGGCGAGACCGCTCGAGTTACTACGCCCCCAACTGAGAG
SLIV_18945DF	GGCTCTTCAAGACCTCTTGAGGAGTTGTAGCCTGCGACCGTTCGACCCGGTACCGGAGTA
SLIV_18945DR	CGATGACGACGCGCCGGTTCACGACGGCACTCACCACCTTACTACGCCCCCAACTGAGAG
SLIV_04375DF	CGCCACCCAGTCAGTCCCCTACGCAAGGGAGGTACGTCCATGTCGACCCGGTACCGGAGTA
SLIV_04375DR	GGACGCGGTCCACCGGGTCCGGCGCGGTGAACCGCTCAGTTCACTACGCCCCCAACTGAGAG
Cl10_DF	CTACGTGGACGAGGAGGAGTCCTTCGTACGGTCACGGTCCTGGTGGTGGTTCGACCCGGTACCGGAGTA
Cl10_DR	CAACGCGCGACATGGCGGGGCAGCAGGAACGGATACGGGCGGCGCAGGATACTACGCCCCCAACTGAGAG

SLIV_10445ChF	ATCACGGCAGAACGGCACTA
SLIV_10445ChR	TCGAATCCTTGGCACGTCAC
SLIV_ 12150ChF	CGATCAGCCTCTACAAACAG
SLIV_ 12150ChR	TTACTCCTCGACGAGCAGCT
SLIV_12280ChF	GAGAGTCGAACGAAGCGAAA
SLIV_12280ChR	TAACCCGCGGATTAGCACGT
SLIV_12285ChF	TGCCGTTCGTACGTTGCTGA
SLIV_12285ChR	TCGCTGCAATCCGTCTCGTG
SLIV_12645ChF	ACCGCTTATCCGGAGCTAAC
SLIV_12645ChR	TCATGACCGAACTCTCCGTC
SLIV_14515ChF	TGCACAACAACGGGCACGGA
SLIV_14515ChR	AAGCGTGCGACCTTCGTGCT
SLIV_18805ChF	ACATCAAGGTGACCAGCTGA
SLIV_18805ChR	TCATGTCTGTCGTCGACGAC
SLIV_19850DisF	ATGACTGACTCGACGCCTTG
SLIV_19850DisR	CTCGTCGAGAGGTCCCTGAA
SLIV_16170ChF	AGCTGCGTCCGTTCCTGAC
SLIV_16170ChR	GATCGGCTACTTCTGCGCC
SLIV_21000ChF	GATCGGAAGGCGGTTGACAT
SLIV_21000ChR	AACACGGCCACCGCCGTAC
SLIV_22925ChF	TGCGCAAGGATTCCGTCGTG
SLIV_22925ChR	ATGGGGACAGAGTTACCCGT
SLIV_33225ChF	TTCGTCGAACCGTTCGTCGA
SLIV_33225ChR	AAGGCGCTTTACGGAGGCTT
SLIV_12885ChF	CCTGGGCCAACCTACGGTT
SLIV_12885ChR	ACTGGCGTGCGCCATCTCA
SLIV_17315ChF	CAAGTGGCTACTCGGAGCGA
SLIV_17315ChR	AGGGTCGTGACTCAGGCCG
SLIV_18945ChF	TACCCGGCTCTTCAAGACCT
SLIV_18945ChR	GGCAGTTCCTGCGCGCACA
SLIV_04375ChF	TCAGTCCCCTACGCAAGGGA
SLIV_04375ChR	AGCGCACTTCGAAATCAAGAC
Cl10_ChF	GCGAGACCGGCTCCGAGA
Cl10_ChR	GTGCGTGATCCACAGCGGA

Supplementary Table 2. List and coordinates of BAC clones of *S. lividans* TK24 genomic BAC library (additional file Data Sheet 2).

Supplementary Table 3. Location and size of *S. lividans* TK24 chromosome regions not covered in the BAC library.

Gap	Not covered CDCs		Coordinates, kb		Gap size, kb
	Start	End	Start	End	
1	*SLIV_00005*	*SLIV_00035*	1	8,299	8299
2	*SLIV_00465*	*SLIV_00530*	92,792	105,462	12,671
3	*SLIV_01050*	*SLIV_01150*	216,848	236,306	19,459
4	*SLIV_03440*	*SLIV_03515*	733,335	752,104	18,770
5	*SLIV_04755*	*SLIV_04840*	1,036,820	1,057,054	20,235
6	*SLIV_04915*	*SLIV_04975*	1,072,692	1,087,759	15,068
7	*SLIV_05615*	*SLIV_05630*	1,251,072	1,255,868	4,797
8	*SLIV_06305*	*SLIV_06425*	1,421,903	1,453,964	3,2062
9	*SLIV_07590*	*SLIV_07635*	1,743,066	1,755,290	12,225
10	*SLIV_09275*	*SLIV_09360*	2,122,758	2,140,777	18,020
11	*SLIV_10300*	*SLIV_10305*	2,381,302	2,381,688	387
12	*SLIV_25190*	*SLIV_25195*	5,637,999	5,641,009	3011
13	*SLIV_25615*	*SLIV_25620*	5,743,339	5,744,223	885
14	*SLIV_33230*	*SLIV_33295*	7,384,843	7,399,115	14,273
15	*SLIV_34200*	*SLIV_34305*	7,583,419	7,602,315	18,897
16	*SLIV_36065*	*SLIV_36070*	7,980,129	7,982,880	2,752
17	*SLIV_36275*	*SLIV_36350*	8,040,486	8,060,079	19,594
18	*SLIV_36605*	*SLIV_36720*	8,120,976	8,145,003	24,028
19	*SLIV_36880*	*SLIV_36975*	8,178,938	8,198,970	20,033
20	*SLIV_37245*	*SLIV_37280*	8,252,267	8,267,231	14,965
21	*SLIV_37405*	*SLIV_37620*	8,300,997	8,345,283	44,287
**total**		**263 CDCs**			**324,718**

Supplementary Table 4. List of sigma factors in *S.lividans* TK24 and *S.ceolicolor* M145 and results of transcriptomics analysis (additional file Data Sheet 3).

Supplementary Table 5. List of strains generated in this study.

	**Strain**	**Deleted gene ID**	***S. ceolicolor* homologue**	**Predicted product**	**BAC clone used for deletion**
1	TK24Δ10445	*SLIV_10445*	*SCO5621*	RNA polymerase sigma factor WhiG	1717 am
2	TK24Δ12150	*SLIV_12150*	*SCO5243*	RNA polymerase sigma factor SigH	1062 am
3	TK24Δ12280	*SLIV_12280*	*SCO5217*	anti-sigma R factor	814 am
4	TK24Δ12285	*SLIV_12285*	*SCO5216*	RNA polymerase sigma factor SigR	1266 am
5	TK24Δ12645	*SLIV_12645*	*SCO5147*	RNA polymerase sigma factor SigR1	625 am
6	-	*SLIV_14515**	*SCO4769*	RNA polymerase sigma factor SigD (ShbA)	595 am
7	TK24Δ18805	*SLIV_18805*	*SCO3892*	RNA polymerase sigma factor SigT	689 hyg
8	TK24Δ19850	*SLIV_19850*	*SCO3690*	SigB regulatory protein RsbQ	1352 hyg
9	TK24Δ16170	*SLIV_16170*	*SCO4452*	RNA polymerase sigma factor SigL1	375 hyg
10	TK24Δ21000	*SLIV_21000*	*SCO3356*	RNA polymerase sigma factor SigE	1178 am
11	TK24Δ22925	*SLIV_22925*	*SCO2954*	RNA polymerase sigma factor SigU	324 hyg
12	TK24Δ33225	*SLIV_33225*	*SCO0942*	RNA polymerase sigma factor SigL2	1209 am
13	TK24Δ12885	*SLIV_12885*	*SCO5100*	GntR family transcriptional regulator	1241 am
14	TK24Δ17315	*SLIV_17315*	*SCO4215*	GntR family regulatory protein	541 am
15	TK24Δ18945	*SLIV_18945*	*SCO3864*	GntR family regulatory protein	877 am
16	TK24Δ04375	*SLIV_04375*	*SCO6974*	GntR family transcriptional regulator	752 am

* - lethal.

Supplementary Table 6. Data table for microtiter-plate strain phenotyping. Relative values for cell-dry-weight (CDW), determined within one hour after maximum scattered light intensity (I_{max}) was reached, maximum specific growth rate (μ_{max}), average projected pellet area ($\bar{A}_{pellet}$) and the cultivation duration, until stationary phase was reached (t_{batch}) are provided for all strains. All values are have been normalized by using the wild-type result in the corresponding cultivation run. n=3 (except CDW: n=2). For the wild-type strain the standard deviation is calculated using all cultivations available, obtained in the separate runs**.**

Strain	CDW		I_{max}		μ_{max}		$\bar{A}_{pellet}$		t_{batch}	
	value	range	value	std	value	std	value	std	value	std
TK24	1.00	0.10	1.00	0.08	1.00	0.05	1,00	0,36	1.00	0.12
TK24Δ10445	1.01	0.02	1.10	0.02	0.94	0.03	0,94		1.00	0.01
TK24Δ12150	0.79	0.00	1.05	0.08	0.78	0.09	0,72		1.01	0.01
TK24Δ12280	0.94	0.04	1.05	0.05	0.74	0.03	0,86		1.01	0.03
TK24Δ12285	0.96	0.02	1.11	0.04	0.85	0.03	1,13		1.01	0.05
TK24Δ12645	0.66	0.03	0.85	0.08	0.69	0.06	1,96		1.21	0.04
TK24Δ18805	0.90	0.04	1.13	0.05	0.93	0.04	0,93		0.95	0.02
TK24Δ19850	0.90	0.12	1.05	0.00	0.89	0.02	0,81		0.94	0.02
TK24Δ16170	1.01	0.00	1.13	0.04	1.01	0.01	0,40		1.00	0.05
TK24Δ21000	0.94	0.03	1.08	0.04	1.02	0.08	0,65		1.11	0.03
TK24Δ22925	0.86	0.02	1.00	0.01	0.89	0.02	0,41		1.07	0.06
TK24Δ33225	1.01	0.00	1.17	0.02	1.05	0.06	0,54		1.01	0.02
TK24Δ12885	0.91	0.01	0.95	0.05	1.10	0.03	0,43		1.06	0.01
TK24Δ17315	0.97	0.01	1.08	0.03	0.99	0.09	0,53		1.03	0.05
TK24Δ18945	0.93	0.01	1.01	0.03	1.13	0.08	0,60		0.97	0.01
TK24Δ04375	1.02	0.01	1.07	0.02	1.06	0.04	0,40		0.96	0.10

Supplementary Table 7. *S. lividans* secretome changes in sigma factor gene deletion strains determined by nanoLC-MSMS (additional file Data Sheet 4).

Proteins identified by mass spectrometric workflow (see Methods). Quantitative values (determined using iBAQ), Sequence coverage for the identified proteins and number of peptides per protein for each biological sample are included.

Table S7 is provided in the additional spreadsheet file:

“Table S7_Proteins identifed by mass spectrometry (Sheet 1)”.

Comparison of WT vs deletion strain for differentially synthesized proteins, for the secreted proteins based on annotation in the SToPS database http://www.stopsdb.eu [1]

“Table S7_ Proteins identifed by mass spectrometry (sheet 2)”.

Supplementary Table 8. Transcription of *S. lividans* TK24 *bldN* orthologue (*SLIV_21180*) and its regulon during cultivation in liquid minimal medium (additional file Data Sheet 5).

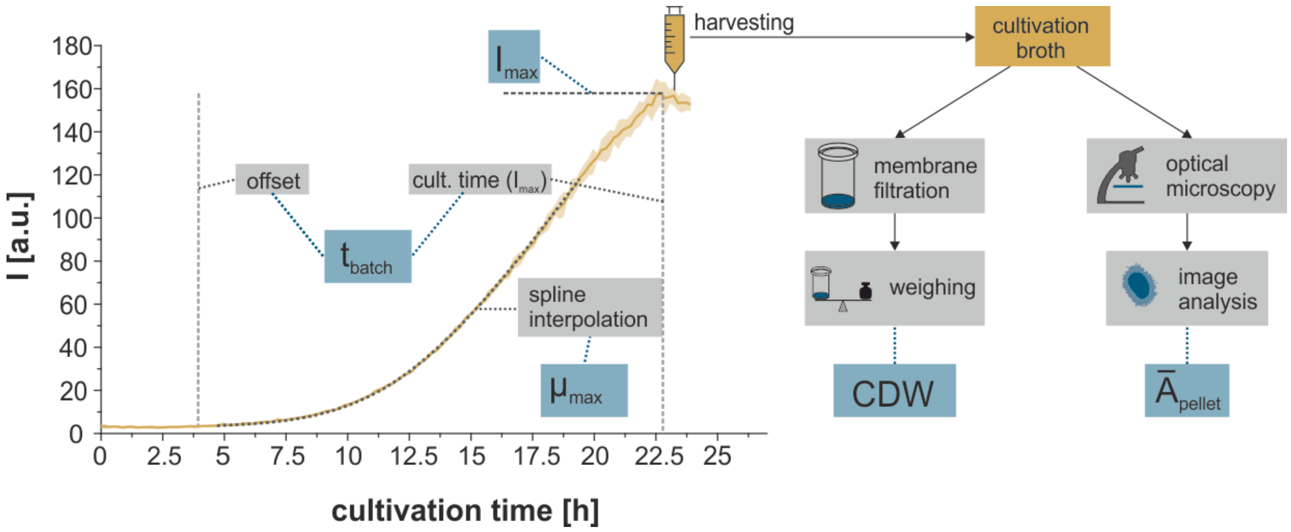

Supplementary Figure 1. Fitness testing of sigma factor deletion strains using microtiter-plate based screening workflow.

Scattered light intensity pattern (TK24, n=3) is provided in the plot (solid orange line: average, semi-transparent background: standard deviation). I_{max} was obtained directly from online signals. t_{batch} was calculated as the cultivation time difference between the fist value, which exceeded the standard the fluctuation by factor 2 and the cultivation time of I_{max}. μ_{max} was estimated by spline approximation as previously published [2]. Two of the three replicate wells were harvested within one hour, after I_{max} was reached. 800 µL of the sampled volume was membrane filtrated (pore size 0.22 µm) and washed in pre-weighted tubes. Cell dry weight (CDW) was determined by drying and weighing the filtrated biomass. Using the remaining sampling volume, light-microscopical images were taken by a Leica DMLB instrument, equipped with a 10x objective (Leica Microsystems, Wetzlar, Germany) and a DCC camera (Thorlabs, Newton, NJ, United States). Pellet identification and image analysis was realized applying an in-house developed Fiji (version 7) based image processing pipeline (in detail described in [3]) to obtain average pellet size ($\bar{A}_{pellet}$).

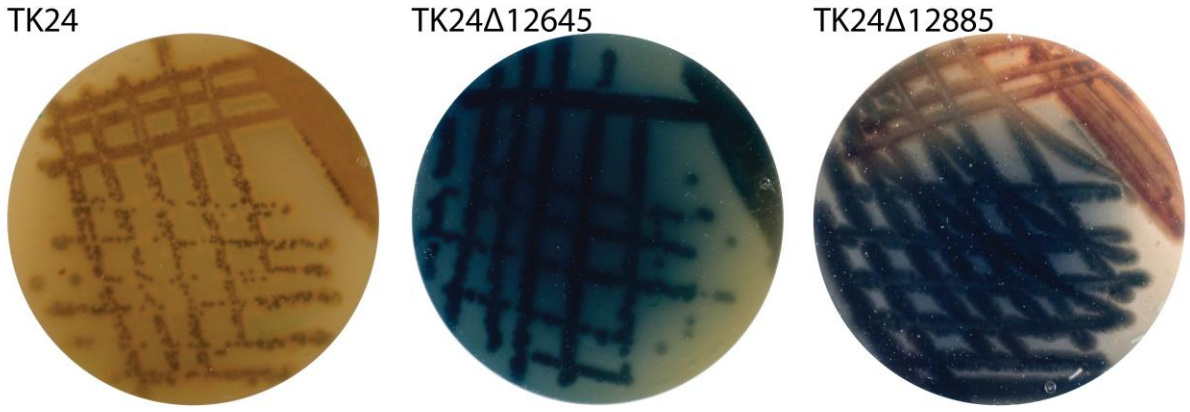

Supplementary Figure 2. Growth of *S. lividans TK24* and mutants lacking *SLIV_12645* and *SLIV_12885* on MS agar plates.

Back view. Strains were grown for 5 days at 28° C. Blue compound corresponds to actinorhodin and red to undecylprodigiosin accumulation.

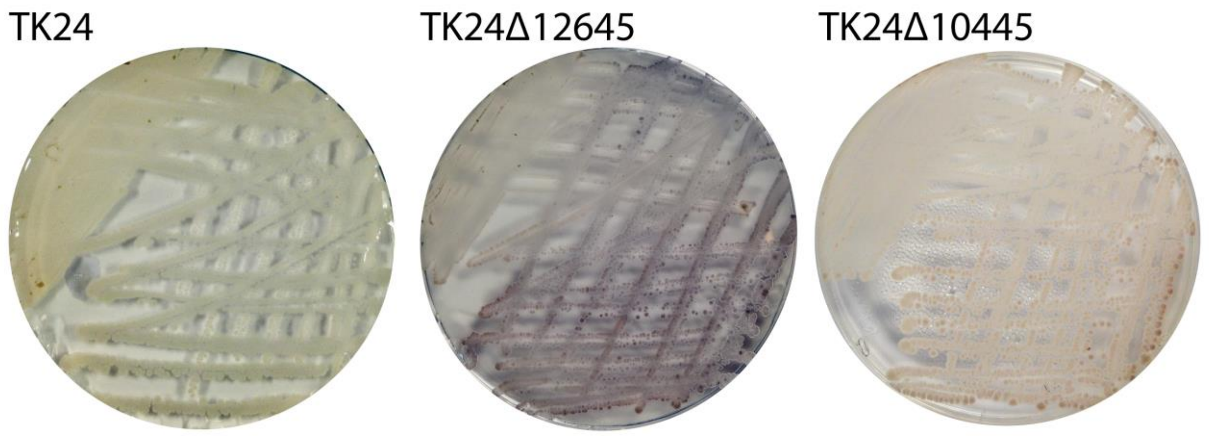

Supplementary Figure 3. Growth of *S. lividans TK24* and mutants lacking *SLIV_12645* and *SLIV_10445* on Minimal medium plates.

Back view. Strains were grown for 5 days at 28° C. Blue compound corresponds to actinorhodin and red to undecylprodigiosin accumulation.

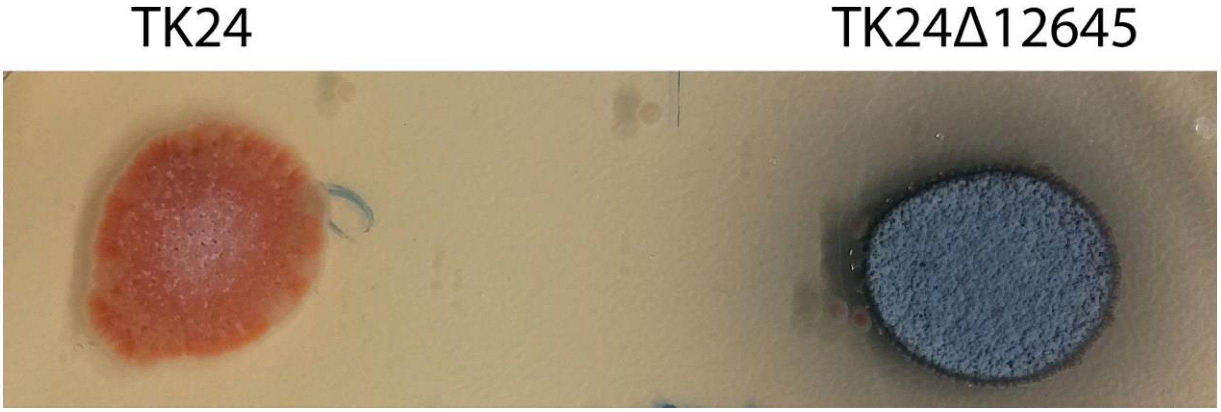

Supplementary Figure 4. Growth of *S. lividans TK24* and mutant lacking *SLIV_12645* on TSB agar plate.

Strains were grown for 5 days at 28° C. Blue compound corresponds to actinorhodin and red to undecylprodigiosin accumulation.

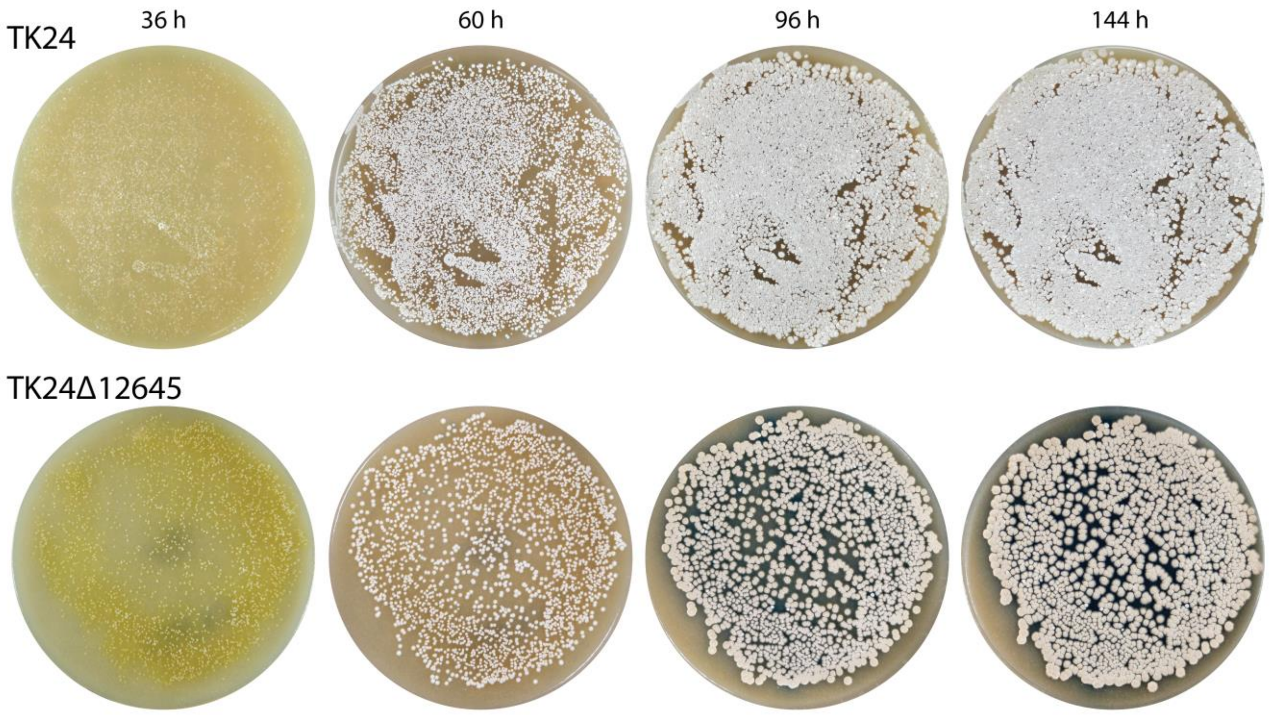

Supplementary Figure 5. Production of different secondary metabolites by *S. lividans* TK24 and TK24Δ12645 at different time points of cultivations on MS agar plates.

Strains were grown at 28° C. Yellow compound (corresponds to coelimycin P1) was ectopically accumulated between 24 and 48 hours of growth. Blue compound corresponds to actinorhodin.

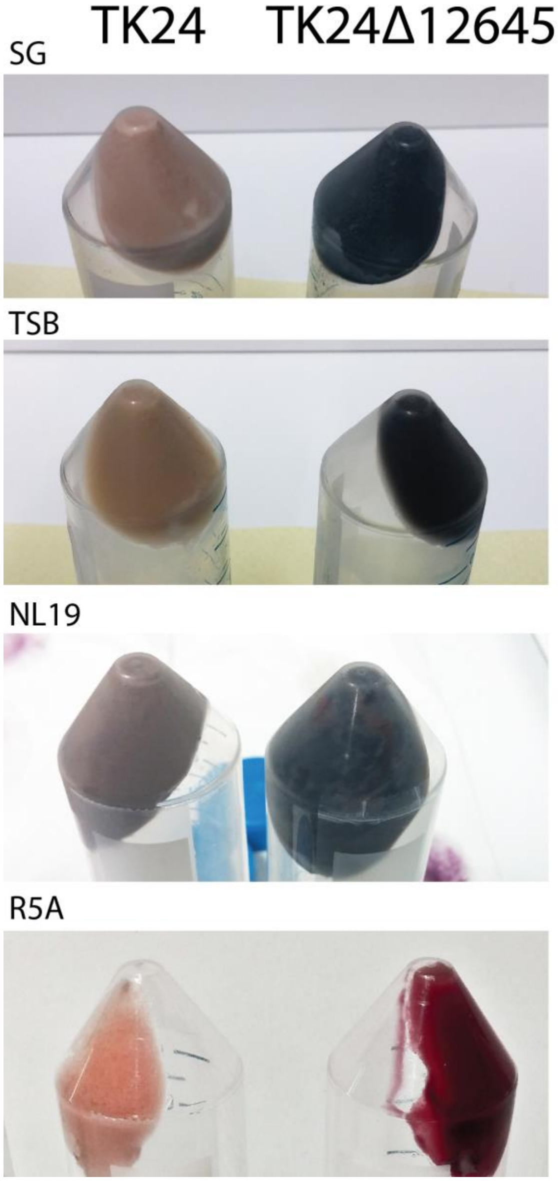

Supplementary Figure 6. Accumulation of secondary metabolites by *S. lividans* TK24 and *SLIV_12645* mutant cultivated in different liquid media.

Strains were cultivated in 50 ml of indicated liquid production media in 500 ml flasks for 5 days at 28° C and 180 rpm. Blue compound corresponds to actinorhodin and red to undecylprodigiosin accumulation.

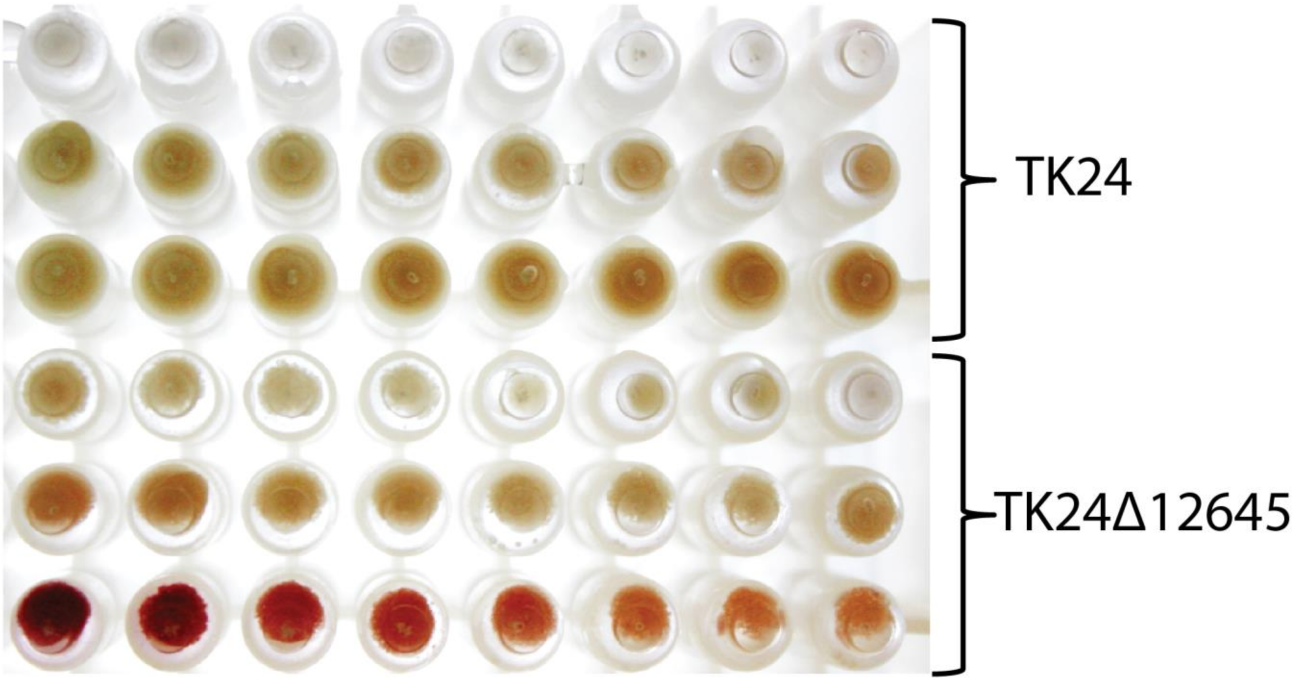

Supplementary Figure 7. Microtiter-plate cultivation of *S. lividans* TK24 and TK24Δ12645 in liquid minimal medium.

Strains were cultivated in microtiter-plate as described in Material and Methods. Red compound corresponds to undecylprodigiosin accumulation.

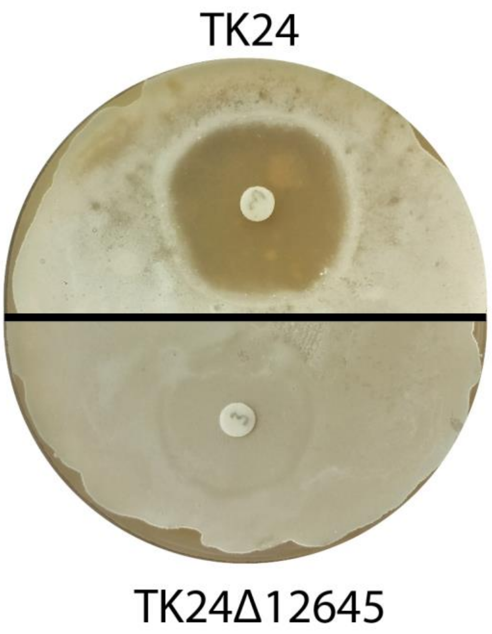

Supplementary Figure 8. Sensitivity of *S. lividans* TK24 and TK24Δ12645 mutant strain to 10 μM of diamide after 5 days of incubation at 28°.

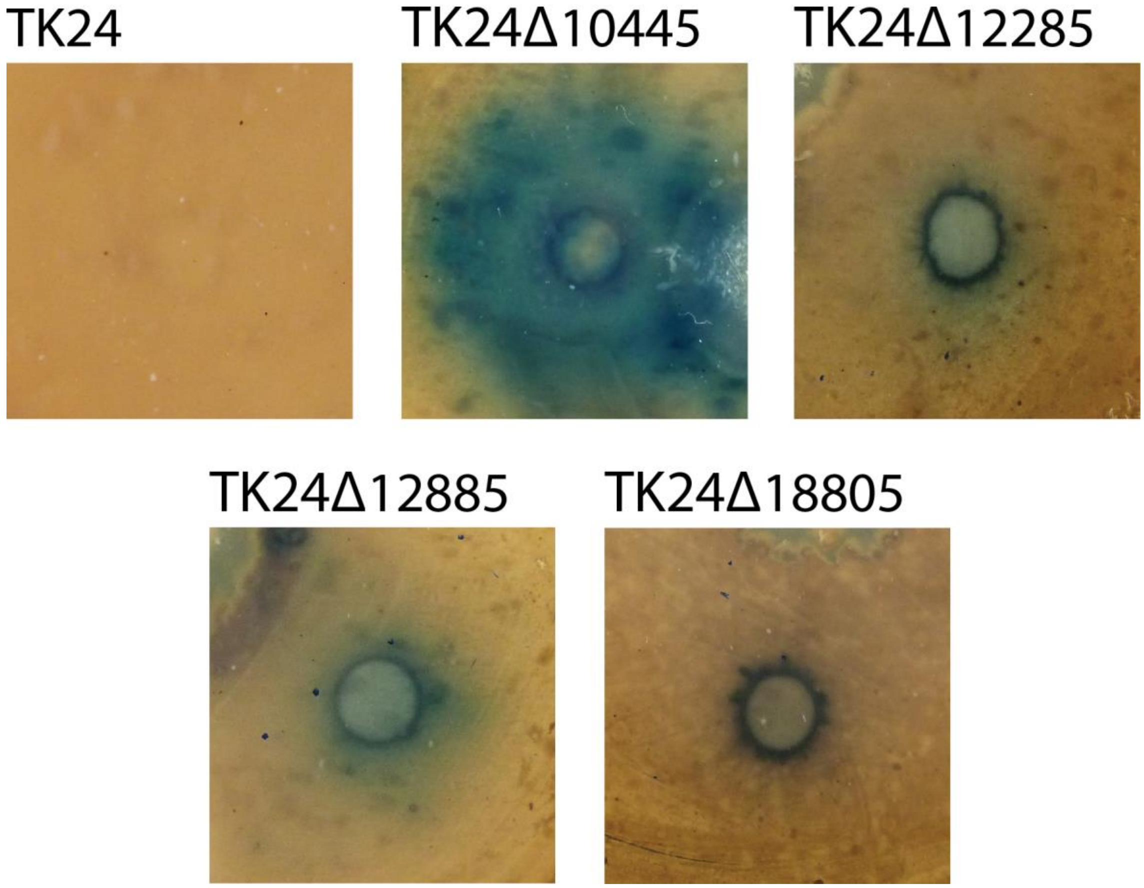

Supplementary Figure 9. Induction of actinorhodin production by hydrogen peroxide in *S. lividans* TK24 mutants lacking sigma factor coding genes.

Paper discs soaked in 10% hydrogen peroxide solution were placed on top of freshly plated spores of corresponding strain plated on MS medium. Plates were incubated at 28° C for 5 days. Blue compound corresponds to actinorhodin.

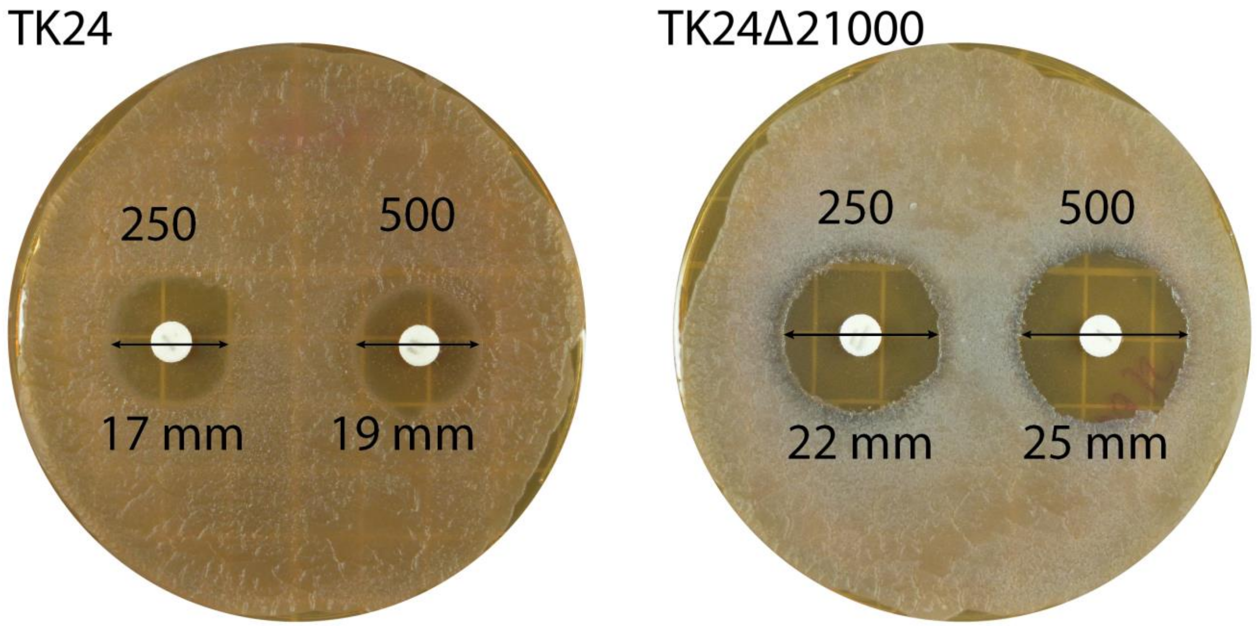

Supplementary Figure 10. Vancomycin sensitivity of *S. lividans* TK24 and TK24Δ21000 mutant grown on TSB agar plates.

Paper discs loaded with 250 or 500 μg of vancomycin, dried and placed on top of freshly plated spores of corresponding strain plated on TSB (or MS – not shown) medium. Plates were incubated at 28° C for 3 days.

References:

1. Tsolis, K.C., et al., *Comprehensive subcellular topologies of polypeptides in Streptomyces.* Microb Cell Fact, 2018. **17**(1): p. 43.
2. Radek, A., et al., *Miniaturized and automated adaptive laboratory evolution: Evolving Corynebacterium glutamicum towards an improved D-xylose utilization.* Bioresource Technology, 2017. **245**: p. 1377-1385.
3. Koepff, J., et al., *Fast and reliable strain characterization of Streptomyces lividans through micro-scale cultivation.* Biotechnol Bioeng, 2017. **114**(9): p. 2011-2022.
